# Nutritional Interventions to Optimize Orthobiologic Therapy Quality in Type 2 Diabetes Mellitus: Molecular Mechanisms and Clinical Framework: A Narrative Review

**DOI:** 10.3390/ijms27093749

**Published:** 2026-04-23

**Authors:** Márcia da Silva Santos, Fábio Ramos Costa, João Protásio Netto, Gabriel Silva Santos, Rubens Martins, Luyddy Pires, André Kruel, Gabriel Azzini, José Fábio Lana

**Affiliations:** 1 Department of Nutritional Sciences, Metropolitan Union of Education and Culture (UNIME), Salvador 42700-000, BA, Brazil; marcinha_mairi@hotmail.com; 2 Department of Orthopedics, FC Sports Traumatology Clinic, Salvador 40296-210, BA, Brazil; fabiocosta123@uol.com.br; 3 Department of Orthopedics and Traumatology, Federal University of Tocantins (UFT), Palmas 77001-090, TO, Brazil; 4 Department of Orthopedics, Brazilian Institute of Regenerative Medicine (BIRM), Indaiatuba 13334-170, SP, Brazil; gabriel1_silva@hotmail.com (G.S.S.); luyddypires@gmail.com (L.P.); kruel.andre@gmail.com (A.K.); drgabriel.azzini@gmail.com (G.A.); josefabiolana@gmail.com (J.F.L.); 5 Regenerative Medicine, Orthoregen International Course, Indaiatuba 13334-170, SP, Brazil; 6 Medical School, Tiradentes University Center, Maceió 57038-800, AL, Brazil; rubensdeandrade@hotmail.com; 7 Medical School, Max Planck University Center (UniMAX), Indaiatuba 13343-060, SP, Brazil; 8 Clinical Research Department, Anna Vitória Lana Institute (IAVL), Indaiatuba 13334-170, SP, Brazil; 9 Medical School, Jaguariúna University Center (UniFAJ), Jaguariúna 13820-000, SP, Brazil

**Keywords:** type 2 diabetes, orthobiologics, platelet-rich plasma, bone marrow aspirate concentrate, mesenchymal stem cells, nutritional interventions, mitochondrial biogenesis, advanced glycation end-products, regenerative medicine, metabolic optimization

## Abstract

Type 2 diabetes mellitus (T2DM) affects approximately 10–25% of patients undergoing orthopedic procedures and is associated with impaired tissue healing, increased complication rates, and reduced responsiveness to orthobiologic therapies, including platelet-rich plasma (PRP), bone marrow aspirate concentrate (BMAC), and mesenchymal stem cell (MSC) preparations. The underlying mechanisms include advanced glycation end-product accumulation, NF-κB-driven chronic inflammation, Nrf2 pathway impairment, mitochondrial dysfunction, and epigenetic diabetic memory, collectively compromising both orthobiologic product quality and the tissue microenvironment. Emerging, predominantly mechanistic evidence suggests that targeted nutritional interventions, including bioactive compounds targeting mitochondrial biogenesis pathways, anti-inflammatory dietary patterns, and specific micronutrients, may modulate these pathological processes and potentially improve orthobiologic outcomes. This narrative review synthesizes evidence from diabetic pathophysiology, orthobiologic outcomes research, and nutritional science to propose a conceptual clinical framework for regenerative medicine optimization in T2DM patients. Critical knowledge gaps are identified, and a research agenda is proposed. The proposed framework, based primarily on mechanistic and preclinical evidence, should be interpreted as a foundation for research prioritization and hypothesis generation rather than as a clinical protocol. Rigorous randomized trials directly evaluating nutritional optimization in orthobiologic therapy for diabetic patients are required before evidence-based recommendations can be established.

## 1. Introduction

Type 2 diabetes mellitus (T2DM) has reached pandemic proportions, affecting over 537 million adults globally, with projections indicating 783 million by 2045 [1]. The intersection of diabetes and musculoskeletal pathology represents a significant clinical challenge, as T2DM patients comprise 10–25% of orthopedic surgery populations and experience substantially higher complication rates [2,3]. Wukich and colleagues established that diabetes adversely affects outcomes across all orthopedic subspecialties, including increased surgical site infections, delayed wound healing, and impaired bone consolidation [4].

The burden of diabetic musculoskeletal complications extends beyond acute surgical outcomes. Meta-analytic evidence demonstrates that diabetic patients have an odds ratio of 2.11 for impaired fracture healing compared to non-diabetic individuals [5], while tendinopathy prevalence is markedly elevated, with 27.5% of diabetic patients experiencing shoulder disorders compared to 5% in the general population [6,7]. A notable 19-year follow-up study confirmed a persistent increased risk of tendon injury in patients with structured diabetes care. This suggests that even well-managed T2DM confers ongoing musculoskeletal vulnerability [8].

Orthobiologic therapies, including platelet-rich plasma (PRP), bone marrow aspirate concentrate (BMAC), and mesenchymal stem cell (MSC) treatments, have emerged as promising interventions for musculoskeletal pathology [9,10,11]. These autologous therapies leverage the body’s regenerative mechanisms through concentrated growth factors, cytokines, and progenitor cells. However, accumulating evidence suggests that diabetic patients may experience attenuated responses to these therapies. Park and colleagues demonstrated in a meta-analysis that diabetic patients undergoing rotator cuff repair had significantly higher retear rates (28.2% vs. 19.3%), with uncontrolled diabetes increasing this risk to 40% [12]. Similarly, Abate et al. found that diabetic patients receiving PRP for tendinopathy achieved less improvement than non-diabetic controls, with HbA1c levels independently predicting poor outcomes [13].

The mechanisms underlying impaired orthobiologic efficacy in diabetes are multifactorial. Hyperglycemia induces mesenchymal stem cell senescence and depletes specific regenerative subpopulations, particularly angiogenic and vasculogenic progenitor subpopulations, including CD34^+^ endothelial progenitor cells and pro-angiogenic mesenchymal stem cell subsets [14,15]. On the other hand, advanced glycation end-products (AGEs) accumulate in tendons, cartilage, and bone, impairing tissue mechanics and cellular function [16,17]. The concept of “diabetic memory” suggests that prior hyperglycemic exposure causes persistent epigenetic modifications that impair progenitor cell function even after glycemic normalization [18,19]. Critically, mitochondrial dysfunction has emerged as a central pathophysiological mechanism linking metabolic dysregulation to impaired regenerative capacity [20,21].

Emerging evidence suggests that nutritional interventions may modulate these pathological mechanisms and potentially optimize orthobiologic quality and therapy outcomes. Montagnino et al. recently proposed that exercise, diet, and supplements could enhance PRP and stem cell therapy efficacy through effects on platelet function, MSC viability, and the tissue microenvironment [22]. This aligns with the growing recognition that patient metabolic status influences regenerative medicine outcomes [23].

This narrative review synthesizes evidence from three previously siloed domains. Rather than a systematic review, this work offers an integrative synthesis with a clinical framework proposal, examining: (1) the pathophysiology of impaired tissue regeneration in T2DM, (2) orthobiologic therapy outcomes in diabetic populations, and (3) nutritional interventions that modulate regenerative pathways, with particular emphasis on mitochondrial biogenesis as an emerging therapeutic target. The integration of these domains has remained relatively underexplored for several reasons: the historical separation of endocrinology and orthopedic subspecialties, the relatively recent emergence of orthobiologics as mainstream interventions, and the traditionally separate research communities investigating nutritional biochemistry versus clinical outcomes. Additionally, the complexity of diabetic pathophysiology, involving multiple interconnected mechanisms, has made translational synthesis challenging. We discuss an integrated clinical framework for optimizing orthobiologic quality in diabetic patients and identify priority research questions to advance this emerging field.

## 2. Pathophysiology of Impaired Regeneration in Type 2 Diabetes

### 2.1. Advanced Glycation End-Products and RAGE Signaling

Advanced glycation end-products (AGEs) are irreversible adducts formed through non-enzymatic glycation of proteins, lipids, and nucleic acids under hyperglycemic conditions. In musculoskeletal tissues, AGEs accumulate in collagen-rich structures, including tendons, ligaments, cartilage, and bone matrix, fundamentally altering tissue biomechanics and cellular function [16,24]. Patel and colleagues demonstrated that AGEs dose-dependently reduce tendon fibroblast proliferation and mitochondrial ATP production, establishing a direct link between glycation and impaired tendon regenerative capacity [17].

The receptor for advanced glycation end-products (RAGE) mediates many pathological effects of AGEs in musculoskeletal tissues. RAGE is expressed on osteoblasts, osteoclasts, chondrocytes, and tenocytes, and its activation triggers intracellular signaling cascades, including NF-κB and MAPK pathways [25,26]. Taguchi and Fukami recently reviewed RAGE signaling in diabetic complications, demonstrating that RAGE activation promotes vascular calcification and impairs tissue repair through sustained inflammatory signaling, particularly via NF-κB activation and increased expression of pro-inflammatory cytokines such as TNF-α, IL-1β, and IL-6 [27]. The AGE-RAGE axis creates a feed-forward loop: RAGE activation increases RAGE expression, amplifying cellular damage in chronically hyperglycemic environments.

In bone, AGE accumulation impairs collagen cross-linking patterns, reducing fracture toughness independent of bone mineral density [28]. Saito and colleagues demonstrated inverse correlations between pentosidine (a well-characterized AGE) and bone mechanical properties, partially explaining the “diabetic bone paradox”, wherein diabetic patients have increased fracture risk despite normal or elevated bone mineral density [24]. These findings have direct implications for orthobiologic therapies targeting bone regeneration, as the tissue microenvironment may limit therapeutic cell function regardless of growth factor concentration.

### 2.2. NF-κB Dysregulation in Skeletal Stem Cells

Nuclear factor kappa-B (NF-κB) is a master regulator of inflammatory responses with critical roles in tissue repair. In normal healing, NF-κB activation occurs transiently during the inflammatory phase, then resolves to permit transition to proliferative and remodeling phases. Ko and colleagues demonstrated, using diabetic mouse models, that diabetes induces persistent NF-κB activation in skeletal stem cells, preventing resolution of inflammation and impairing bone regeneration [29]. Pharmacological NF-κB inhibition rescued skeletal stem cell function and improved bone regeneration in these models, establishing a mechanistic link between inflammatory dysregulation and impaired diabetic healing. Whether these preclinical findings translate directly to human orthobiologic outcomes remains to be established.

This inflammatory dysregulation has profound implications for orthobiologic therapy. Mesenchymal stem cells transplanted into a diabetic microenvironment encounter persistent inflammatory signals that may redirect their differentiation toward fibrotic rather than regenerative phenotypes. Baker and colleagues demonstrated that constitutive NF-κB activation in metabolic tissues promotes insulin resistance and systemic inflammation, creating a systemic milieu unfavorable for regeneration [30]. These findings suggest that anti-inflammatory nutritional interventions may improve orthobiologic efficacy by attenuating persistent inflammatory signaling, although this extrapolation from murine models to clinical nutritional practice requires prospective validation.

### 2.3. Oxidative Stress and Nrf2/Keap1 Pathway Impairment

The nuclear factor erythroid 2-related factor 2 (Nrf2) pathway represents the master regulator of cellular antioxidant defense. Under normal conditions, Keap1 sequesters Nrf2 in the cytoplasm, targeting it for proteasomal degradation. Oxidative stress causes Keap1 conformational changes that release Nrf2 to translocate to the nucleus and activate antioxidant response element (ARE)-driven genes. Long and colleagues from the Zhang laboratory reported that Nrf2 knockout diabetic mice exhibit severely delayed wound healing, while pharmacological Nrf2 activation with sulforaphane or cinnamaldehyde accelerates diabetic wound closure [31], highlighting Nrf2 as a potentially relevant molecular target. The therapeutic modulation of this pathway through dietary interventions in orthobiologic patients, however, remains to be established in clinical settings.

Subsequent work by Soares and colleagues demonstrated that aberrant Keap1 overexpression in diabetic tissues prevents appropriate Nrf2 nuclear translocation, and that siRNA-mediated Keap1 knockdown restores regenerative capacity in high-glucose fibroblasts [32,33]. These findings establish the Nrf2/Keap1 axis as a therapeutic target for enhancing tissue regeneration in diabetes. Dodson et al. recently provided a comprehensive update on Nrf2 in diabetes, noting the complexity of this pathway and cautioning that while Nrf2 activation is generally protective, constitutive hyperactivation may have unintended consequences [34].

The Nrf2 pathway is particularly relevant to orthobiologic therapy because progenitor cell function depends on redox homeostasis. Bone marrow-derived MSCs exposed to hyperglycemic environments exhibit impaired Nrf2 signaling, contributing to their reduced regenerative potential [35]. Nutritional Nrf2 activators, including sulforaphane from cruciferous vegetables, curcumin, and resveratrol, may therefore improve the function of both endogenous and transplanted stem cells in diabetic patients.

These observations derive predominantly from in vitro and preclinical models; direct clinical evidence for sulforaphane, curcumin, or resveratrol in orthobiologic populations is currently unavailable. Whether Nrf2 pathway modulation through dietary means translates to meaningful improvement in progenitor cell function and orthobiologic outcomes in diabetic patients warrants investigation in adequately powered prospective studies.

### 2.4. Mitochondrial Dysfunction and Impaired Biogenesis

Mitochondrial dysfunction has emerged as a central pathophysiological mechanism in diabetic tissue impairment, with direct relevance to orthobiologic quality and efficacy. Hyperglycemia induces mitochondrial fragmentation, reduces oxidative phosphorylation capacity, and impairs the biogenesis of new mitochondria through suppression of peroxisome proliferator-activated receptor gamma coactivator 1-alpha (PGC-1α), the master regulator of mitochondrial biogenesis [20,36]. Patti and colleagues demonstrated that PGC-1α expression is reduced by 25–50% in skeletal muscle of T2DM patients, contributing to metabolic inflexibility and impaired tissue regeneration [37].

The implications for orthobiologic therapy are profound. Mesenchymal stem cells require robust mitochondrial function for proliferation, differentiation, and paracrine activity [38]. Mitochondrial dysfunction in the diabetic milieu impairs both MSC potency and platelet function, potentially compromising the quality of autologous orthobiologic products despite adequate cell counts.

Beyond biogenesis, mitochondrial dynamics, the continuous processes of fission and fusion, are essential for maintaining mitochondrial quality control. OPA1 (optic atrophy 1) and mitofusins (Mfn1/2) promote fusion, while Drp1 (dynamin-related protein 1) mediates fission. Diabetic tissues exhibit dysregulated dynamics with excessive fission, leading to fragmented mitochondrial networks with impaired function [39]. Emerging preclinical evidence suggests that nutritional and pharmacological interventions targeting mitochondrial dynamics may help restore balanced fission–fusion cycles. Additionally, mitochondrial transfer from healthy cells to damaged tissues has been demonstrated to restore cellular function, suggesting a contributory role in tissue regeneration [40,41]. These findings establish mitochondrial optimization as a rational target for enhancing orthobiologic quality in diabetic patients.

An emerging aspect of mitochondrial quality control is selective mitophagy mediated by the PINK1/Parkin pathway. Under normal conditions, healthy mitochondria maintain low levels of PINK1 (PTEN-induced kinase 1) through continuous import of the PINK1 protein into the inner mitochondrial membrane followed by its proteolytic degradation. When mitochondria become damaged and lose membrane potential, PINK1 accumulates on the outer membrane and recruits the E3 ubiquitin ligase Parkin, tagging dysfunctional mitochondria for autophagic degradation. This quality control mechanism is impaired in diabetic tissues, leading to accumulation of damaged mitochondria that generate excessive reactive oxygen species and perpetuate cellular dysfunction. Importantly, urolithin A, a gut microbiome-derived compound discussed in Section 4, activates mitophagy through the PINK1/Parkin pathway, providing a mechanistic rationale for its inclusion in mitochondrial optimization protocols for diabetic patients undergoing orthobiologic therapy.

It should be noted that evidence for urolithin A and other mitochondrial optimization compounds in the orthobiologic context derives entirely from indirect preclinical findings; no randomized trials have evaluated these agents in diabetic patients undergoing orthobiologic therapy. The mechanistic rationale described above should therefore be understood as hypothesis-generating rather than as a basis for current clinical recommendations.

### 2.5. Mesenchymal Stem Cell Dysfunction and Diabetic Memory

Perhaps the most challenging aspect of diabetic regenerative impairment is the phenomenon of “metabolic memory,” also termed “diabetic memory.” Giacco and Brownlee’s foundational work established that transient hyperglycemia induces persistent cellular dysfunction through mitochondrial superoxide overproduction and subsequent epigenetic modifications [18]. These changes persist even after glucose normalization, explaining the continued progression of diabetic complications in patients achieving glycemic control.

Natarajan’s 2020 Edwin Bierman Award Lecture provided compelling evidence that epigenetic modifications, including DNA methylation changes and histone post-translational modifications, persist for 16–17 years after the end of intensive glycemic control periods [19]. Reddy and colleagues further characterized these mechanisms, demonstrating that hyperglycemia induces both activating and repressive histone modifications at genes regulating inflammation and vascular function [42].

For orthobiologic therapy, diabetic memory has profound implications. Rennert, Januszyk, and colleagues at Stanford used single-cell transcriptomics to demonstrate that diabetes selectively depletes angiogenic adipose-derived stem cell subpopulations [43]. These changes are not reversed by acute glycemic normalization. This suggests that autologous cell therapies may have intrinsically impaired potency in diabetic patients. This has led some authors to recommend allogeneic over autologous stem cell sources for diabetic recipients [44]. High glucose also accelerates MSC senescence through autophagy upregulation, reducing the available pool of functional progenitor cells [14,15].

A critical question for clinical practice is whether nutritional interventions can reverse these persistent epigenetic modifications or merely attenuate their functional consequences. Current evidence suggests that nutritional and lifestyle interventions primarily act through the latter mechanism, modulating downstream pathways (AMPK, SIRT1, Nrf2, PGC-1α) rather than erasing epigenetic marks directly. However, emerging research indicates that certain interventions may influence epigenetic machinery: SIRT1 activators affect histone acetylation patterns, while compounds like sulforaphane can modify histone deacetylase activity. The extent to which these effects translate to meaningful reversal of diabetic memory in human tissues remains an important area for investigation. From a practical standpoint, nutritional optimization likely provides benefit through pathway modulation even without complete epigenetic reversal, supporting its inclusion in prehabilitation protocols while acknowledging that some regenerative impairment may persist.

### 2.6. Platelet Dysfunction in Diabetes

Platelets from diabetic patients exhibit multiple functional abnormalities relevant to PRP therapy. Vinik and colleagues established that diabetic platelets show hyperreactivity, increased adhesion molecule expression, and altered growth factor content [45]. Paradoxically, this hyperreactivity may not translate to enhanced PRP efficacy. Karina et al. made the intriguing observation that PRP from diabetic donors contains higher VEGF concentrations than non-diabetic PRP, yet clinical outcomes remain inferior [46]. This paradox suggests that the tissue microenvironment, rather than growth factor concentration, may be the rate-limiting factor for PRP efficacy in diabetes.

Additional platelet abnormalities in diabetes include loss of prostacyclin and nitric oxide sensitivity, insulin resistance affecting platelet function, and elevated markers of platelet activation, including P-selectin and platelet factor 4 [47,48]. These changes create a prothrombotic state that may impair the delicate balance between inflammation and resolution required for optimal tissue healing. Understanding these platelet-specific changes is essential for developing strategies to optimize PRP preparation and application in diabetic patients.

### 2.7. SIRT1 Reduction and Impaired Macrophage Polarization

Sirtuin 1 (SIRT1) is an NAD+-dependent deacetylase with critical roles in metabolic regulation, stress resistance, and longevity. Diabetic tissues, particularly endothelial cells, skeletal muscle, adipose tissue, and wound tissue, exhibit marked SIRT1 reduction, contributing to impaired angiogenesis and wound healing [49]. Li and colleagues demonstrated that pharmacological SIRT1 activation with SRT1720 improves wound healing in diabetic mice by protecting endothelial cells from oxidative stress-induced apoptosis [49]. Berlanga-Acosta proposed that combined “metabolic memory” and “senescence memory” create persistent regenerative impairment through epigenetic mechanisms involving SIRT1 [50].

Macrophage polarization from pro-inflammatory M1 to regenerative M2 phenotypes is essential for normal tissue repair. Wang and Graves comprehensively reviewed macrophage plasticity in diabetic wound healing, demonstrating that NF-κB promotes M1 polarization while TGF-β/Smad signaling promotes M2 transition [51]. In diabetes, persistent inflammatory signals maintain M1 predominance, delaying the transition to regenerative phases. Al-Mulla and colleagues showed impaired TGF-β signaling in diabetic wounds, mechanistically linking growth factor dysfunction to macrophage polarization defects [52]. Recent work has identified epigenetic regulation of macrophage phenotype, with M1 genes showing hypomethylation and M2 genes hypermethylation in diabetic contexts [53].

### 2.8. Integration of Pathophysiological Mechanisms

**Figure 1:**
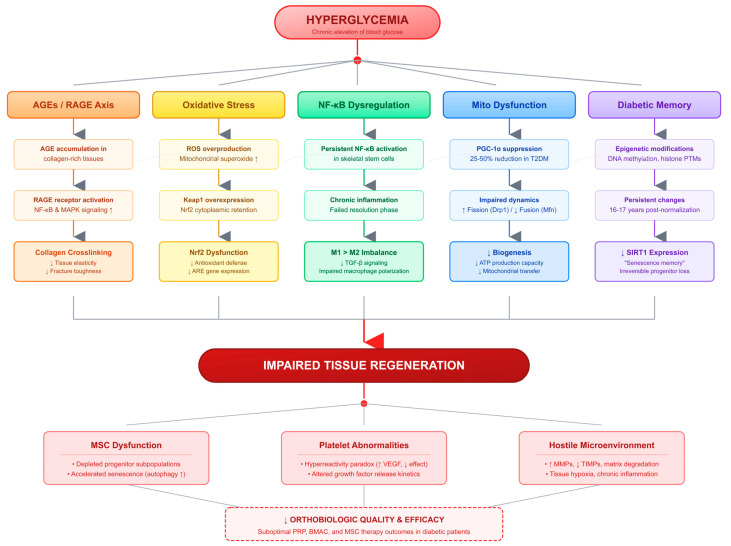
Pathophysiological mechanisms of impaired tissue regeneration in type 2 diabetes mellitus. Hyperglycemia initiates five interconnected pathways: (1) AGEs/RAGE signaling leading to collagen crosslinking and matrix dysfunction [17,24]; (2) oxidative stress with paradoxical Nrf2 dysfunction reducing antioxidant defenses [31,32]; (3) persistent NF-κB activation driving chronic inflammation and M1/M2 macrophage imbalance [29,51]; (4) mitochondrial dysfunction with impaired PGC-1α-mediated biogenesis and altered fission/fusion dynamics [20]; and (5) epigenetic “diabetic memory” causing persistent cellular dysfunction through DNA methylation and histone modifications [18,19]. These pathways converge on impaired tissue regeneration, manifesting as MSC dysfunction, platelet abnormalities, and a hostile tissue microenvironment that collectively reduce orthobiologic therapy efficacy. Abbreviations: AGEs, advanced glycation end-products; RAGE, receptor for AGEs; Nrf2, nuclear factor erythroid 2-related factor 2; NF-κB, nuclear factor kappa-B; PGC-1α, peroxisome proliferator-activated receptor gamma coactivator 1-alpha; MSC, mesenchymal stem cell.

The pathophysiological mechanisms described above do not operate in isolation but rather form an interconnected network of dysfunction. Mitochondrial impairment (Section 2.4) directly contributes to diabetic memory (Section 2.5) through persistent ROS generation that drives epigenetic modifications. AGE accumulation (Section 2.1) activates RAGE signaling that feeds into NF-κB dysregulation (Section 2.2), while simultaneously impairing mitochondrial function. Oxidative stress from Nrf2/Keap1 impairment (Section 2.3) exacerbates mitochondrial damage and promotes AGE formation. SIRT1 reduction (Section 2.7) impairs both mitochondrial biogenesis (through PGC-1α) and epigenetic regulation, linking metabolic dysfunction to persistent gene expression changes (Figure 1).

**Figure 2:**
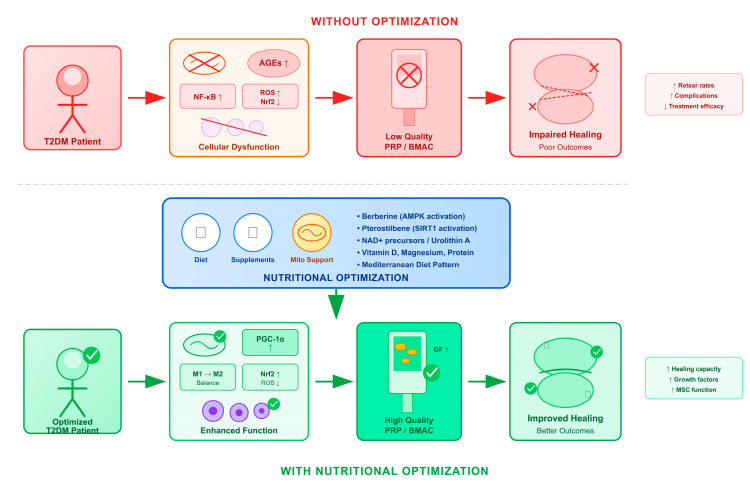
Graphical abstract. Conceptual overview of the proposed framework, in which nutritional optimization modulates the systemic and tissue-level environment before orthobiologic therapy in type 2 diabetes mellitus. The upper panel depicts the unoptimized diabetic state, associated with impaired tissue regeneration related to hyperglycemia, oxidative stress, chronic inflammation, mitochondrial dysfunction, and epigenetic “diabetic memory.” The lower panel illustrates the targeted effects of nutritional optimization, integrating an anti-inflammatory dietary pattern (Mediterranean-style), correction of prevalent micronutrient deficiencies, and bioactive compounds acting on AMPK, SIRT1, NAD+, mitophagy and Nrf2 pathways, which together are proposed to improve the biological substrate for subsequent orthobiologic intervention. The schematic synthesizes the mechanisms and interventions detailed in the subsequent figures and should be interpreted as a conceptual guide rather than as a clinical protocol.

Beyond the mechanisms specific to hyperglycemia, obesity itself represents an independent driver of orthobiologic impairment through chronic low-grade inflammation, termed ‘meta-inflammation’ [54,55]. Excess adipose tissue elevates circulating cytokines, including IL-6 and TNF-α, which impair MSC proliferative capacity, increase cellular senescence, and compromise the therapeutic potential of autologous orthobiologic products [54]. Fernandes and Rodeo recently proposed the concept of Metabolic Optimization Before Orthobiologic Therapies (MOBOT), emphasizing that host metabolic health (including factors such as obesity, physical inactivity, inflammaging, sleep disturbances, and gut dysbiosis) which may be as important as product-related variables in determining orthobiologic outcomes [54]. This broader perspective reinforces the need for comprehensive metabolic assessment extending beyond glycemic control alone. This crosstalk creates feed-forward loops that amplify regenerative impairment: for example, mitochondrial ROS activates NF-κB, which promotes inflammation and further mitochondrial damage. Understanding these interconnections is important for intervention design, as targeting a single pathway may be insufficient without addressing related mechanisms. The nutritional interventions discussed in Section 4 target multiple nodes in this network simultaneously. Berberine activates AMPK while reducing inflammation, pterostilbene activates SIRT1 while providing antioxidant effects, and the Mediterranean diet pattern modulates inflammation, oxidative stress, and metabolic function concurrently (Figure 2).

## 3. Orthobiologic Therapy Outcomes in Diabetic Patients

### 3.1. Platelet-Rich Plasma for Diabetic Wound Healing

The largest body of evidence for PRP in diabetic populations comes from diabetic foot ulcer (DFU) studies. Multiple meta-analyses have evaluated PRP efficacy for DFU, consistently demonstrating benefit over standard care. Xu and colleagues analyzed 15 randomized controlled trials encompassing 1010 patients and found a relative risk of 1.53 for complete healing with PRP compared to conventional treatment [56]. Meznerics et al. conducted the most comprehensive analysis to date, including 29 RCTs with 2198 wounds, reporting an odds ratio of 5.32 for wound closure with PRP [57]. Su and colleagues confirmed these findings, reporting significantly shorter healing times with PRP intervention [58].

The Cochrane Collaboration’s systematic review by Martinez-Zapata et al., while more conservative in its conclusions, still found a relative risk of 1.22 for DFU healing with PRP, though evidence quality was rated as low [59]. Del Pino-Sedeño and colleagues additionally noted reduced adverse events with PRP therapy (RR 0.80), supporting its safety profile [60]. Despite these positive findings, it is important to recognize that DFU represents a distinct clinical entity from musculoskeletal pathology, and extrapolation to tendon, cartilage, or bone healing requires caution.

### 3.2. Platelet-Rich Plasma for Diabetic Tendinopathy

Evidence for PRP efficacy specifically in diabetic musculoskeletal conditions is more limited. Abate and colleagues conducted the most informative study, retrospectively comparing 60 diabetic patients to 60 non-diabetic controls receiving PRP for Achilles or patellar tendinopathy [13]. Diabetic patients achieved significantly less improvement on validated outcome scores (−2.76 VISA score difference, *p* = 0.003), and HbA1c levels independently predicted poor outcomes (OR 1.16 per unit increase). This study provides supportive evidence that diabetes attenuates PRP efficacy for tendon pathology, though its retrospective design and single-center nature warrant confirmation in prospective studies.

The finding that metabolic control (HbA1c) correlates with treatment response suggests that optimization of glycemic status before orthobiologic therapy may improve outcomes. This hypothesis aligns with established evidence that perioperative hyperglycemia adversely affects surgical outcomes and has been proposed as a modifiable risk factor for regenerative therapy [61].

### 3.3. Bone Marrow Aspirate Concentrate and Mesenchymal Stem Cell Therapies

Evidence for BMAC and MSC therapy outcomes specifically in diabetic musculoskeletal patients is sparse. Most MSC therapy trials in diabetes have focused on glycemic control rather than tissue regeneration. Kashbour and colleagues’ meta-analysis of 13 RCTs demonstrated that MSC therapy reduces HbA1c by 0.72% and insulin requirements by 14.5 units in diabetic patients, but these studies did not assess musculoskeletal outcomes [62]. Wang et al. similarly reported improved C-peptide levels with MSC transplantation [63].

For diabetic foot ulcers, Tong and colleagues’ recent meta-analysis of 24 studies with 1321 patients found that stem cell therapy significantly improves healing rates compared to standard care [64]. The analysis compared different stem cell sources and found benefits across multiple cell types. However, significant heterogeneity in protocols, cell sources, and outcome measures limits definitive conclusions. Yu and colleagues reviewed stem cell therapy for DFU and recommended consideration of allogeneic sources given the impaired function of autologous diabetic-derived cells [44].

An important consideration for BMAC therapy in diabetics is the documented impairment of hematopoietic stem cell mobilization. Çelik and colleagues reported mobilization failure rates of 15.6% in diabetic patients compared to 6.4% in non-diabetic controls [65]. This may reflect underlying bone marrow niche dysfunction and suggests that diabetic patients may require modified BMAC harvest protocols or alternative cell sources.

While human clinical data on nutritional optimization for BMAC/MSC quality in diabetic musculoskeletal applications remain limited, preclinical studies provide supportive mechanistic evidence. Animal models have demonstrated that caloric restriction improves bone marrow stem cell engraftment and regenerative capacity, while high-fat diet-induced metabolic dysfunction impairs MSC differentiation potential. Studies in diabetic rodent models show that berberine pretreatment improves bone marrow-derived cell function and enhances wound healing outcomes. Similarly, resveratrol and pterostilbene supplementation in diabetic animals have been shown to preserve MSC proliferative capacity and reduce senescence markers. These preclinical findings, while requiring clinical translation and validation, support the biological plausibility of nutritional optimization strategies for enhancing autologous cell therapy quality in diabetic patients.

### 3.4. The Tissue Microenvironment Hypothesis

**Figure 3:**
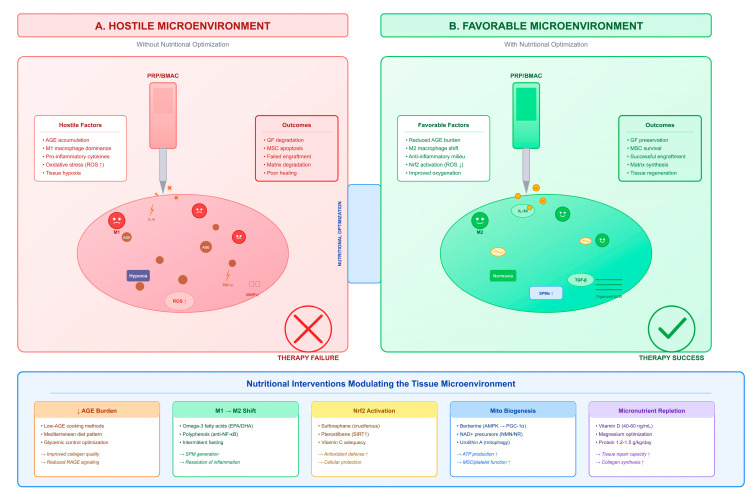
The tissue microenvironment hypothesis: hostile versus favorable conditions for orthobiologic therapy. (**A**) In the unoptimized diabetic state (left panel), the tissue microenvironment presents multiple barriers: accumulated AGEs, M1 macrophage dominance, elevated pro-inflammatory cytokines (IL-6, TNF-α), oxidative stress (elevated ROS), tissue hypoxia, and elevated MMP activity that degrades growth factors. (**B**) Following nutritional optimization (right panel), the microenvironment shifts toward regeneration-permissive conditions: reduced AGE burden, M2 macrophage polarization with anti-inflammatory factors (IL-10, TGF-β), Nrf2-mediated antioxidant defense restoration, improved oxygenation, and balanced MMP/TIMP ratios preserving growth factors. The lower panel summarizes the five categories of nutritional interventions (AGE burden reduction, M1-to-M2 polarization, Nrf2 activation, mitochondrial biogenesis, and micronutrient repletion) that modulate the transition from panel A to panel B. Abbreviations: AGEs, advanced glycation end-products; IL, interleukin; TNF-α, tumor necrosis factor-alpha; ROS, reactive oxygen species; MMP, matrix metalloproteinase; TGF-β, transforming growth factor-beta; Nrf2, nuclear factor erythroid 2-related factor 2; TIMP, tissue inhibitor of metalloproteinase.

The paradox of increased growth factor content in diabetic PRP yet reduced clinical efficacy [46] supports the tissue microenvironment hypothesis: that the recipient tissue environment, rather than the therapeutic product composition, is the primary determinant of orthobiologic success in diabetes. The diabetic microenvironment features persistent hyperglycemia, chronic M1-predominant inflammation, tissue hypoxia, and elevated matrix metalloproteinase activity, creating conditions that may degrade growth factors, misdirect cellular differentiation, and prevent the establishment of regenerative cascades regardless of the quality of the orthobiologic product delivered (Figure 3).

This concept has been independently validated by Fernandes and Rodeo, who observed that despite methodological rigor in product design, inconsistent patient response remains a persistent challenge, suggesting that host-related factors play an underrecognized role [54]. Their MOBOT framework proposes that screening and managing modifiable systemic factors (including insulin resistance, chronic inflammation, sarcopenia, and poor sleep) are essential components of optimizing the biological environment for orthobiologic success.

Recent advances in biomaterials have attempted to address microenvironment limitations through scaffolds that release anti-inflammatory factors, hydrogels that sequester AGEs, or oxygen-releasing microspheres to address hypoxia. However, nutritional interventions offer a complementary approach: by systemically modifying the metabolic environment before therapy, the tissue milieu receiving orthobiologic treatment may be more conducive to regeneration. This concept underlies the clinical framework proposed in this review.

The distinction between optimizing orthobiologic product quality versus the recipient microenvironment has important clinical implications. Interventions targeting product quality (for example, improving platelet mitochondrial function or enhancing MSC potency through pre-harvest supplementation) primarily affect the concentrated cells and growth factors delivered during therapy. In contrast, microenvironment optimization (e.g., reducing tissue AGE burden, shifting macrophage polarization, restoring antioxidant defenses) affects the tissue bed receiving the therapy. Nutritional interventions likely act through both mechanisms: systemic supplementation improves the metabolic health of cells harvested for autologous therapy while simultaneously modifying the tissue environment. This dual action may explain why comprehensive metabolic optimization could offer advantages over approaches targeting only the product or the environment. Future research should attempt to dissect these contributions through study designs that isolate product-level effects (e.g., comparing optimized versus non-optimized autologous PRP in the same patient) from systemic effects (e.g., comparing outcomes in metabolically optimized versus non-optimized recipients receiving standardized allogeneic products).

## 4. Nutritional Modulation of Regenerative Pathways

### 4.1. Macronutrient Considerations

#### 4.1.1. Protein Intake and Muscle–Tendon Health

Adequate protein intake is fundamental for tissue repair and has particular relevance for diabetic patients at risk of sarcopenia. The American Diabetes Association recommends 1.0–1.5 g/kg/day protein intake for T2DM patients without diabetic kidney disease, with higher intakes appropriate for those regaining lean mass [66]. The European Association for the Study of Diabetes (EASD) and the Diabetes Nutrition Study Group (DNSG) similarly emphasize individualized protein recommendations [67]. Diabetic sarcopenia affects approximately 18% of T2DM patients, with a 2–3-fold higher risk compared to non-diabetic populations [68].

Leucine, a branched-chain amino acid, directly activates the mTORC1 pathway through mechanisms involving S6K1 and 4E-BP1 phosphorylation [69,70]. This pathway is critical for protein synthesis and may support tendon and muscle regeneration. However, evidence for leucine supplementation specifically improving diabetic tissue healing is limited. Leenders et al. found that 6-month leucine supplementation (7.5 g/day) did not significantly improve muscle mass or strength in elderly T2DM patients. This suggests that protein quality and overall metabolic status may be more important than single amino acid supplementation [71]. Fusaro and colleagues’ systematic review on nutrition and tendon health supports protein adequacy but does not identify optimal protein sources for tendinopathy management [72].

#### 4.1.2. Carbohydrate Quality and AGE Reduction

Dietary advanced glycation end-products (dAGEs) represent a modifiable contributor to the total AGE burden. Baye and colleagues’ meta-analysis of 17 RCTs demonstrated that low-AGE diets improve insulin sensitivity (mean difference −1.3 HOMA-IR) and reduce LDL cholesterol [73]. de Courten et al. showed that in a well-designed crossover RCT that a 2-week low-AGE diet increases insulin sensitivity in overweight individuals [74]. Mark and colleagues confirmed that high-AGE diets increase HOMA-IR, supporting the bidirectional relationship between dietary AGEs and metabolic dysfunction [75].

Practical strategies for reducing dietary AGE intake include using moist cooking methods (steaming, poaching, stewing) rather than high-heat dry methods (grilling, frying, roasting), choosing minimally processed foods, and limiting consumption of highly browned or charred items. These modifications may complement pharmacological approaches targeting the AGE-RAGE axis and could theoretically improve the tissue microenvironment for orthobiologic therapy, though direct evidence for this application is lacking.

An additional practical strategy for glycemic optimization with relevance to the prehabilitation period is meal sequencing, specifically consuming protein- and fiber-rich foods before carbohydrates within each meal. Shukla and colleagues demonstrated in a series of studies that this ‘carbohydrate-last’ eating pattern significantly reduces postprandial glucose and insulin excursions in patients with type 2 diabetes [76,77]. Ma et al. demonstrated that protein preloads effectively reduce postprandial glycemia through effects on gastric emptying and gut hormone release [78]. This simple behavioral modification requires no supplementation or dietary restriction and can be readily integrated into prehabilitation protocols as a complementary strategy to reduce glycemic variability and AGE formation during the optimization phase.

#### 4.1.3. Omega-3 Fatty Acids

Omega-3 polyunsaturated fatty acids, particularly eicosapentaenoic acid (EPA) and docosahexaenoic acid (DHA), exert anti-inflammatory effects through multiple mechanisms, including specialized pro-resolving mediator (SPM) generation and modulation of macrophage phenotype [79]. These effects are theoretically relevant to optimizing the tissue environment for orthobiologic therapy. Soleimani and colleagues demonstrated in a randomized trial that flaxseed oil supplementation (1000 mg/day for 12 weeks) reduced diabetic foot ulcer size and improved insulin resistance [80].

For musculoskeletal applications specifically, Sandford and colleagues conducted an RCT of omega-3 supplementation for rotator cuff-related shoulder pain [81]. Importantly, the primary outcome (shoulder pain and disability at 12 months) did not differ significantly between groups; the early benefit observed at 3 months (64% vs. 42% improvement) was not sustained. This null primary outcome represents the most directly relevant available evidence for omega-3 in a musculoskeletal context, and the general anti-inflammatory evidence base should not be extrapolated to orthobiologic applications without direct supporting data.

### 4.2. Micronutrient Considerations

#### 4.2.1. Vitamin D

Vitamin D deficiency is highly prevalent in orthopedic populations and is associated with adverse outcomes. Llombart and colleagues’ meta-analysis demonstrated that vitamin D insufficiency increases mortality risk in hip fracture patients (OR 1.24), with severe deficiency conferring even greater risk (OR 2.08) [82]. Kong et al. found that vitamin D supplementation at doses of 800–1000 IU/day reduces fracture risk (RR 0.87) and fall risk (RR 0.91) [83]. The foundational review by Holick established vitamin D’s role in musculoskeletal health and defined optimal serum levels [84].

However, the VITAL trial, the largest vitamin D supplementation study to date, found that 2000 IU/day did not significantly reduce fracture incidence in vitamin D-replete adults [85]. This finding emphasizes that supplementation benefits are likely confined to deficient populations. Given the high prevalence of vitamin D deficiency in diabetic patients and those with musculoskeletal pathology, screening and correction of deficiency before orthobiologic therapy are rational, with target serum 25(OH)D levels of 40–60 ng/mL proposed for optimal musculoskeletal function.

#### 4.2.2. Magnesium

Hypomagnesemia affects approximately 30% of diabetic patients and is associated with insulin resistance and impaired glycemic control [86]. Simental-Mendía and colleagues’ meta-analysis of 22 RCTs demonstrated that magnesium supplementation improves insulin sensitivity (WMD −0.67 HOMA-IR) [87]. A more recent pooled analysis of 24 RCTs by Xu and colleagues confirmed benefits for glucose control with magnesium supplementation in T2DM [86].

For bone health, Farsinejad-Marj and colleagues’ systematic review found positive correlations between dietary magnesium intake and hip/femoral bone mineral density [88]. Magnesium supplementation in T2DM patients is most appropriately framed as correction of a well-documented deficiency state rather than as a proactive regenerative intervention. While magnesium deficiency has established adverse effects on insulin signaling and bone metabolism, there is no direct evidence linking supplementation-mediated repletion to improved orthobiologic outcomes. Screening for and correcting documented hypomagnesemia before orthobiologic therapy is clinically reasonable; routine supplementation in magnesium-replete patients cannot be recommended for this indication on current evidence.

#### 4.2.3. Zinc and Vitamin C

Zinc is essential for wound healing through roles in cell proliferation, immune function, and collagen synthesis. The Cochrane systematic review by Moore and colleagues evaluated nutritional interventions for diabetic foot ulcers, finding very low-to-low quality evidence for zinc supplementation [89]. Lin and colleagues reviewed zinc’s mechanisms in wound healing, noting its importance in inflammatory regulation and epithelialization [90]. While zinc deficiency is more prevalent in diabetic populations and correction is rational, current evidence does not support routine supplementation in zinc-replete individuals for tissue healing.

Vitamin C serves as an essential cofactor for prolyl and lysyl hydroxylase, enzymes required for collagen synthesis. Table 1 summarizes the recommended nutritional interventions, dosages, and evidence levels for orthobiologic optimization in diabetic patients. DePhillipo and colleagues systematically reviewed vitamin C supplementation for musculoskeletal injuries, noting its established role in collagen metabolism and wound healing [91]. Shaw et al. demonstrated that vitamin C-enriched gelatin supplementation before intermittent activity augments collagen synthesis markers, suggesting a practical peri-exercise or peri-therapy application [92]. Given the higher prevalence of deficiency in diabetic populations, vitamin C adequacy should be ensured before procedures requiring robust collagen synthesis.

### 4.3. Bioactive Compounds and Pathway Modulators

The evidence supporting the following interventions varies considerably, ranging from preclinical studies and mechanistic data to early-phase clinical trials and, in some cases, randomized controlled trials.

#### 4.3.1. SIRT1 Activators: Pterostilbene over Resveratrol

Given the documented reduction in SIRT1 in diabetic tissues [49], pharmacological or nutritional SIRT1 activation represents an attractive therapeutic target. Milne and colleagues reported in *Nature* that small-molecule SIRT1 activators such as SRT1720, SRT2104, and SRT3025 are 1000-fold more potent than resveratrol and improve metabolic parameters in rodent models of diabetes [109]. However, clinical translation of synthetic SIRT1 activators has been limited by off-target effects and lack of efficacy in human trials.

Among natural SIRT1 activators, pterostilbene (trans-3,5-dimethoxy-4′-hydroxystilbene) has demonstrated more favorable pharmacokinetic properties than resveratrol. Pterostilbene demonstrates approximately 4-fold higher oral bioavailability (80% vs. 20%) and a 7.5-fold longer half-life (105 min vs. 14 min) compared to resveratrol, resulting in substantially higher tissue concentrations at equivalent doses [110,111]. McCormack and colleagues demonstrated that pterostilbene effectively activates SIRT1 and improves metabolic parameters in animal models of diabetes [112]. Huang and colleagues showed that stilbene compounds promote diabetic wound healing through SIRT1-FOXO1-c-Myc signaling, restoring angiogenesis in diabetic mice [97]. Doses of pterostilbene used in studies typically range from 100 to 250 mg/day, achieving measurable tissue levels in available studies, although clinical relevance in orthobiologic contexts remains to be established.

Safety considerations for pterostilbene supplementation merit discussion, particularly in the context of diabetic patients who may be using multiple medications. Human studies at doses up to 250 mg/day have reported a favorable safety profile with no serious adverse events. However, pterostilbene may have mild effects on LDL cholesterol at higher doses, which should be monitored in patients with dyslipidemia. Potential drug interactions are largely theoretical but include possible additive effects with antiplatelet agents (relevant for patients on aspirin) and theoretical interactions with cytochrome P450 substrates. Given the polypharmacy common in T2DM patients, pterostilbene should be introduced with awareness of concurrent medications and appropriate monitoring. The absence of long-term human safety data (>6 months) represents a limitation that should be communicated to patients.

#### 4.3.2. AMPK Activators: Berberine: A Hypothesis for Investigation in Exercise-Prehabilitation Contexts

AMP-activated protein kinase (AMPK) is a master metabolic regulator that promotes glucose uptake, fatty acid oxidation, and mitochondrial biogenesis while inhibiting lipogenesis and protein synthesis through mTOR suppression. Hardie’s authoritative review established AMPK as a therapeutic target for diabetes and related metabolic disorders [98]. While metformin is the most widely used AMPK activator, emerging mechanistic evidence raises the hypothesis (requiring dedicated clinical investigation) that berberine may offer a complementary or alternative profile in the specific context of exercise-based prehabilitation for regenerative medicine.

A critical consideration for orthobiologic optimization is the interaction between pharmacological agents and exercise-induced adaptations. The MASTERS trial demonstrated that metformin blunts skeletal muscle hypertrophy and strength gains in response to resistance training in older adults [99]. Walton and colleagues further showed that metformin inhibits exercise-induced mitochondrial adaptations, potentially counteracting the benefits of prehabilitation protocols [100]. In contrast, berberine activates AMPK through a different mechanism and does not appear to block exercise-induced adaptations [113,114].

Berberine (900–1500 mg/day in divided doses) demonstrates comparable glycemic efficacy to metformin while offering additional benefits, including lipid modulation and anti-inflammatory effects [93,115]. Importantly for orthobiologic applications, berberine preserves the ability of exercise to induce PGC-1α and promote mitochondrial biogenesis. However, clinicians should be aware that high-dose berberine may increase atrogin-1 expression, potentially affecting muscle protein balance, and should monitor patients accordingly [94].

The mechanistic contrast between berberine and metformin regarding exercise-induced adaptations is highlighted as a hypothesis for clinical investigation. Any clinical decision regarding pharmacotherapy modification in T2DM patients falls outside the scope of this narrative review and requires individualized evaluation by the responsible prescriber. The discussion of berberine presented here is included for its mechanistic relevance and does not constitute a recommendation for clinical use, medication substitution, or dose modification of any established therapy.

#### 4.3.3. Nrf2 Activators

Given the established role of Nrf2 impairment in diabetic healing defects [33,34,35,36], nutritional Nrf2 activation is mechanistically attractive. Senger and colleagues compared the potencies of dietary Nrf2 activators, finding sulforaphane (from cruciferous vegetables) approximately 13.5-fold more potent than curcumin at inducing NQO1, a canonical Nrf2 target gene [95]. Cuadrado and colleagues comprehensively reviewed therapeutic targeting of the Nrf2/Keap1 pathway, noting both opportunities and challenges, including the need for tissue-specific activation [96].

Practical application of Nrf2 activation through diet involves regular consumption of cruciferous vegetables (broccoli, Brussels sprouts, cabbage, cauliflower) and spices, including turmeric. Sulforaphane content is highest in broccoli sprouts and can be enhanced by combining raw vegetables with myrosinase-containing foods. While direct evidence for dietary Nrf2 activation improving orthobiologic outcomes is lacking, the strong preclinical evidence for Nrf2’s role in diabetic healing supports this as a promising research direction.

#### 4.3.4. Mitochondrial Biogenesis and Dynamics Modulators

**Figure 4:**
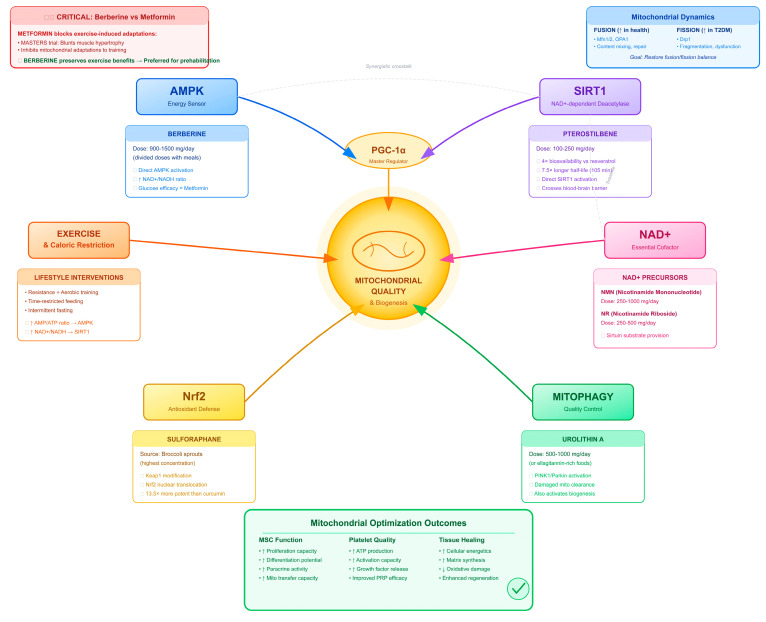
Nutritional targets for mitochondrial optimization in type 2 diabetes. Central hub diagram illustrating PGC-1α as the master regulator of mitochondrial biogenesis, with six major intervention pathways: (1) AMPK activation via berberine (900–1500 mg/day); (2) SIRT1 activation via pterostilbene (100–250 mg/day); (3) NAD+ precursor supplementation via NMN (250–1000 mg/day) or NR; (4) mitophagy activation via urolithin A (500–1000 mg/day) through the PINK1/Parkin pathway; (5) Nrf2 activation via sulforaphane from dietary sources; and (6) exercise combined with caloric restriction/time-restricted feeding. Abbreviations: PGC-1α, peroxisome proliferator-activated receptor gamma coactivator 1-alpha; AMPK, AMP-activated protein kinase; SIRT1, sirtuin 1; NAD+, nicotinamide adenine dinucleotide; NMN, nicotinamide mononucleotide; NR, nicotinamide riboside; PINK1, PTEN-induced kinase 1; Nrf2, nuclear factor erythroid 2-related factor 2. Note: Doses shown represent suggested ranges based on available literature; optimal dosing for orthobiologic optimization has not been established in clinical trials.

Building on the mitochondrial mechanisms detailed in Section 2.4, several nutritional agents targeting PGC-1α-mediated biogenesis merit consideration beyond the AMPK and SIRT1 activators discussed above (Figure 4).

NAD+ precursors, including nicotinamide mononucleotide (NMN) and nicotinamide riboside (NR), have demonstrated emerging evidence for metabolic and mitochondrial benefits in early-phase clinical studies [116]. Yoshino and colleagues showed that NMN improves insulin sensitivity and muscle mitochondrial function in prediabetic women [117]. NAD+ is essential for sirtuin activity, linking NAD+ precursor supplementation to SIRT1-mediated benefits. Doses reported in studies typically range from 250 to 500 mg/day for NR and 250–1000 mg/day for NMN, though optimal dosing for orthobiologic optimization remains undefined.

Urolithin A, a gut microbiome-derived metabolite of ellagitannins, activates mitophagy, the selective degradation of damaged mitochondria, thereby improving overall mitochondrial quality. As discussed in Section 2.4, urolithin A acts through the PINK1/Parkin pathway to enhance selective clearance of dysfunctional mitochondria. Andreux and colleagues conducted a randomized trial demonstrating that urolithin A (500–1000 mg/day for 4 weeks) upregulates mitochondrial gene expression and improves cellular respiration in the skeletal muscle of older adults [104]. Liu and colleagues showed that urolithin A also activates mitochondrial biogenesis through SIRT1 and PGC-1α pathways [118]. This dual action, promoting both mitophagy and biogenesis, suggests a potential role for urolithin A in improving the quality of autologous cell populations used in orthobiologic preparations, although this remains to be validated in clinical contexts.

Coenzyme Q10 (CoQ10) serves as an essential electron carrier in the mitochondrial respiratory chain. Supplementation (100–300 mg/day) may improve mitochondrial function, particularly in the context of statin use, which depletes endogenous CoQ10 [101]. While direct evidence for CoQ10 improving orthobiologic outcomes is lacking, its established safety profile and theoretical rationale support consideration in comprehensive mitochondrial optimization protocols.

#### 4.3.5. Alpha-Lipoic Acid

Alpha-lipoic acid (ALA) is a potent antioxidant with established efficacy for diabetic peripheral neuropathy. The ALADIN study demonstrated that intravenous ALA (600 mg/day for 3 weeks) significantly reduces neuropathy symptoms [102]. The 4-year NATHAN 1 trial showed that ALA may prevent worsening of neuropathy, though it did not reverse established disease [103]. Meta-analyses support oral ALA (600–1800 mg/day) for symptomatic improvement [119].

While ALA’s primary evidence base relates to neuropathy rather than tissue regeneration, its antioxidant and AMPK-activating properties [98] suggest potential benefits for optimizing the tissue environment before orthobiologic therapy. ALA’s favorable safety profile and established use in diabetic populations make it a reasonable consideration, particularly in patients with concurrent neuropathic symptoms.

Collectively, these bioactive compounds target key pathways implicated in diabetic regenerative impairment. However, current evidence is largely derived from preclinical studies or early-phase clinical trials, and their specific impact on orthobiologic therapy outcomes remains to be established. These interventions should therefore be interpreted as hypothesis-generating and require validation in well-designed clinical studies.

### 4.4. Polypharmacy and Drug–Supplement Interactions

Patients with T2DM frequently use multiple medications, creating the potential for interactions with the nutritional supplements discussed above. Clinicians implementing comprehensive optimization protocols should be aware of the following considerations:

Berberine may interact with medications metabolized by cytochrome P450 enzymes (CYP3A4, CYP2D6), potentially affecting levels of statins, calcium channel blockers, and certain antidepressants. Berberine also has additive hypoglycemic effects with sulfonylureas and insulin, requiring glucose monitoring and potential dose adjustments. Concurrent use with metformin may increase gastrointestinal side effects.

Pterostilbene and resveratrol may have mild antiplatelet effects, warranting caution in patients on anticoagulants or dual antiplatelet therapy. These compounds may also interact with CYP1A2 and CYP3A4 substrates.

NAD+ precursors (NMN, NR) have limited documented drug interactions but may theoretically affect medications whose metabolism involves NAD+-dependent enzymes.

Omega-3 fatty acids at high doses (>3 g/day) may potentiate anticoagulant effects and should be used cautiously with warfarin or direct oral anticoagulants.

Vitamin D supplementation at high doses may cause hypercalcemia, particularly in patients taking thiazide diuretics or calcium supplements. Magnesium may reduce the absorption of bisphosphonates, fluoroquinolones, and tetracyclines if taken concurrently.

Given these potential interactions, clinicians implementing optimization protocols are advised to: (1) conduct a comprehensive medication review before initiating optimization protocols; (2) introduce supplements in a staggered manner with monitoring for adverse effects; (3) communicate with all prescribing providers; and (4) prefer food-based interventions (Mediterranean diet, cruciferous vegetables) over supplements when feasible.

## 5. Dietary Patterns and Regenerative Capacity

### 5.1. Mediterranean Diet

The Mediterranean diet, characterized by high intake of vegetables, fruits, whole grains, legumes, nuts, olive oil, and fish, with moderate wine consumption and limited red meat, has robust evidence for cardiometabolic benefits in diabetes. Esposito and colleagues’ meta-analysis found that Mediterranean diet adherence reduces HbA1c by 0.30–0.47% and decreases diabetes incidence by 19–23% [120]. The PREDIMED trial (corrected and republished in 2018) demonstrated cardiovascular risk reduction with a Mediterranean diet supplemented with extra-virgin olive oil or nuts [121]. Papadaki et al. confirmed these findings in a more recent meta-analysis [122], while Schwingshackl and colleagues’ network meta-analysis ranked the Mediterranean diet among the most effective dietary approaches for glycemic control [105].

The mechanisms underlying Mediterranean diet benefits include polyphenol-mediated NF-κB suppression, fiber fermentation to short-chain fatty acids (SCFAs) that activate GPR41/43 receptors and stimulate GLP-1 release, and replacement of pro-inflammatory saturated fats with anti-inflammatory omega-3 and monounsaturated fatty acids. These mechanisms align with the pathophysiological targets identified for optimizing orthobiologic therapy in diabetes: anti-inflammatory effects, improved insulin sensitivity, enhanced antioxidant capacity, and support for mitochondrial function.

### 5.2. Caloric Restriction and Stem Cell Function

Caloric restriction (CR) has profound effects on stem cell function that may be relevant to orthobiologic therapy optimization. Cerletti and colleagues at Harvard demonstrated that 40% CR for 12 weeks increases skeletal muscle satellite cell transplant efficiency 4-fold in mice [106]. The mechanisms involve suppression of mTORC1 in the stem cell niche, leading to enhanced stem cell quiescence and regenerative potential upon activation. Maharajan and colleagues reviewed CR effects on stem cells across tissues, noting consistent benefits through niche modulation and metabolic reprogramming [107].

Translation of chronic CR to clinical orthobiologic optimization is challenging, given the difficulty of sustaining significant caloric restriction in humans. However, the mechanistic insights support the hypothesis that metabolic modulation before stem cell harvest or administration may enhance therapeutic potency. Short-term preoperative caloric restriction or fasting protocols (discussed below) may be more practical applications of this principle.

### 5.3. Intermittent Fasting and Regenerative Capacity

Intermittent fasting (IF) offers a more practical approach to achieving the regenerative benefits of caloric restriction. Mihaylova and colleagues reported that 24 h fasting doubles intestinal stem cell regenerative capacity through fatty acid oxidation and PPAR activation in murine models [108]. This study established that metabolic state profoundly influences stem cell function and suggested that timed nutritional interventions could enhance regenerative therapies.

More recently, Imada and colleagues (also from the Yilmaz laboratory) published in *Nature* the important finding that it is the post-fast refeeding period, rather than fasting itself, that drives regeneration through mTORC1 activation and polyamine synthesis [123]. Critically, this study also demonstrated that refeeding after fasting increases tumor formation in animal models with oncogenic mutations. This finding has important implications for clinical translation: while fasting–refeeding cycles may enhance regeneration, caution is warranted in patients with known malignancy or high cancer risk. The balance between regenerative enhancement and potential tumorigenic risk requires careful consideration in protocol development.

### 5.4. Low-Carbohydrate and Ketogenic Diets

Low-carbohydrate and ketogenic diets have gained interest for diabetes management due to their effects on glycemic control. Choi and colleagues’ meta-analysis found that ketogenic diets reduce fasting blood glucose by 1.29 mmol/L, HbA1c by 1.07%, and HOMA-IR by 0.46 [124]. However, Choy and Louie’s meta-analysis of 11 RCTs found no significant difference at 2 years compared to control diets, questioning long-term advantages [125]. Parry-Strong and colleagues similarly concluded that while low-carbohydrate diets may reduce HbA1c and triglycerides, they offer limited advantage over other strategies [126].

The potential relevance of ketogenic diets to orthobiologic optimization lies in the signaling properties of ketone bodies, particularly β-hydroxybutyrate (βOHB), which has emerged as a signaling metabolite beyond its role as an energy substrate. Shimazu and colleagues identified that βOHB functions as an endogenous inhibitor of class I histone deacetylases, increasing expression of FOXO3A and MT2 (metallothionein 2) to protect against oxidative stress [127]. Newman and Verdin provided an authoritative review of βOHB signaling, noting inhibition of the NLRP3 inflammasome and reduction in IL-1β [128]. Puchalska and Crawford updated this evidence, emphasizing βOHB’s roles in health and disease beyond metabolism [129]. Han and colleagues demonstrated that βOHB delays vascular senescence through HDAC inhibition and p53 β-hydroxybutyrylation [130].

These anti-inflammatory, anti-senescence, and antioxidant properties of βOHB align with the pathophysiological targets for diabetic tissue healing optimization. Whether achieving therapeutic βOHB levels through diet can enhance orthobiologic quality in diabetic patients is an important question for future research.

Important safety considerations apply to ketogenic diet implementation in T2DM patients, particularly those using glucose-lowering medications. Patients taking SGLT2 inhibitors (empagliflozin, dapagliflozin, canagliflozin) face an increased risk of euglycemic diabetic ketoacidosis when combining these medications with very low-carbohydrate diets, as SGLT2 inhibitors promote ketogenesis independent of glycemic status. This potentially serious complication may present with normal or only mildly elevated glucose levels, delaying recognition. Patients on insulin or sulfonylureas require dose adjustments to prevent hypoglycemia when initiating ketogenic diets. Given these considerations, ketogenic diets for orthobiologic optimization should only be undertaken with close medical supervision, appropriate medication adjustments, and patient education regarding warning signs. For many patients, moderate carbohydrate restriction or time-restricted eating may offer a safer approach to achieving metabolic benefits without the risks associated with sustained ketosis. However, no clinical trials have yet tested ketogenic interventions in the context of orthobiologic therapies.

### 5.5. Dietary Inflammatory Index

The Dietary Inflammatory Index (DII) provides a validated method for characterizing the inflammatory potential of dietary patterns [131]. Meta-analyses have consistently associated higher (more pro-inflammatory) DII scores with increased T2DM risk and poorer glycemic control. A meta-analysis of 48 studies encompassing 1.7 million participants found that each 1-point DII increase confers 13% higher odds of T2DM [132]. Hariharan and colleagues confirmed associations between high DII, obesity, and metabolic dysregulation [133]. NHANES analyses have demonstrated positive correlations between DII and fasting glucose, HbA1c, and HOMA-IR [134,135].

The DII may serve as a practical tool for assessing and modifying dietary patterns before orthobiologic therapy. Moving patients from pro-inflammatory to anti-inflammatory dietary patterns could theoretically improve the tissue microenvironment for regenerative interventions, though direct evidence for this application is needed.

### 5.6. Gut Microbiome and the Gut–Bone Axis

Emerging evidence identifies gut microbiota dysbiosis as a modifiable factor influencing musculoskeletal regeneration and orthobiologic therapy outcomes. The gut–bone axis represents a key regulatory pathway linking intestinal microbial communities to skeletal homeostasis through immune modulation, stem cell activity, and metabolic signaling [54]. Recent evidence demonstrates that patients with osteoarthritis exhibit altered gut and joint microbiomes, with reduced diversity and pro-inflammatory taxa, highlighting the connection between dysbiosis and joint degeneration [54].

Dysbiosis interferes with MSC proliferation, differentiation, and immunomodulatory capacity. In germ-free animal models, the absence of microbiota causes stem cell dysfunction that is reversible upon microbiota reconstitution [54]. Dysbiosis also reduces the production of short-chain fatty acids (SCFAs), particularly butyrate, which regulates osteoblast and osteoclast activity and modulates immune responses, supporting bone formation through the gut–bone axis [54]. Furthermore, dysbiosis impairs calcium and vitamin D absorption while altering estrogen and parathyroid hormone metabolism, further compromising bone regeneration.

In murine fracture models, probiotic supplementation improved IL-17F expression, upregulated bone matrix genes (Col1, Runx2), and increased bone volume and strength, particularly when administered before injury [54]. Systematic reviews and meta-analyses in rodent models confirm that probiotics enhance bone mineral density and volume through anti-inflammatory effects, improved gut barrier integrity, and increased SCFA production [54]. However, human studies directly linking microbiota modulation to improved orthobiologic outcomes are still lacking.

For diabetic patients undergoing orthobiologic therapy, gut microbiome optimization through high-fiber, plant-rich diets promoting microbial diversity, targeted probiotic supplementation, and polyphenol intake may represent an additional strategy to enhance the systemic environment for regenerative procedures. The SDIMMMER framework proposed by Lana and colleagues incorporates microbiome assessment as one of seven essential domains for comprehensive metabolic profiling in regenerative medicine patients [55]. While standardized protocols for microbiome modulation in this context are still developing, addressing dysbiosis may complement the nutritional interventions discussed in preceding sections.

## 6. Clinical Framework for Nutritional Optimization

The framework presented in this section is a conceptual, integrative proposal designed to organize existing mechanistic and indirect clinical evidence into a translatable structure. It is not intended as a prescriptive protocol; individual patient factors, clinical judgment, and the absence of direct randomized evidence in this specific context require that all elements be adapted accordingly. The sections below outline potential domains of intervention and their rationale, pending prospective validation.

### 6.1. Preoperative Nutritional Assessment

Nutritional assessment before orthobiologic therapy should be considered as part of comprehensive patient evaluation. Validated screening tools including, the Nutritional Risk Screening 2002 (NRS-2002), predict postoperative complications, with odds ratios of 3.13 for general complications and 2.88 for infections [136]. Ozkalkanli and colleagues found NRS-2002 superior to the Subjective Global Assessment (SGA) for predicting orthopedic surgery complications [137]. Cheung et al.’s network meta-analysis compared multiple screening tools, supporting NRS-2002 or MNA-SF for initial screening [138]. The Global Leadership Initiative on Malnutrition (GLIM) criteria provide standardized diagnostic criteria following positive screening [139].

For diabetic patients receiving orthobiologic therapy, a comprehensive metabolic assessment may include: HbA1c (target < 8% for elective procedures), serum 25(OH)D (target 40–60 ng/mL), serum magnesium (replete if deficient), inflammatory markers (hsCRP) to characterize baseline inflammatory status, and consideration of skin autofluorescence measurement to assess tissue AGE burden. This assessment should occur 8–12 weeks before planned therapy to allow time for optimization.

Skin autofluorescence (SAF) measurement, while offering the advantage of non-invasive tissue AGE assessment, has limited availability in many clinical settings. More accessible alternatives for estimating AGE burden include: serum pentosidine and carboxymethyl-lysine (CML) levels, which can be measured by commercial laboratories; estimated AGE intake based on dietary questionnaires and food frequency assessments; duration and severity of diabetes exposure (diabetes duration × mean HbA1c provides a rough index); and the presence of microvascular complications, which correlate with tissue AGE accumulation. While these alternatives lack the direct tissue assessment provided by SAF, they can inform clinical decision-making regarding the likely AGE burden and the intensity of optimization required. As SAF technology becomes more widely available and affordable, its incorporation into routine preoperative assessment may become feasible.

Beyond metabolic and nutritional parameters, comprehensive pre-therapy assessment should address additional modifiable factors that influence orthobiologic outcomes. Sleep quality should be screened using validated instruments, as sleep deprivation disrupts stem cell self-renewal, migration, and differentiation, inducing epigenetic changes that reduce clonal diversity and promote pro-inflammatory myeloid lineages [54]. Sarcopenia assessment using grip strength testing, the chair stand test, and calf circumference measurement provides additional prognostic information for regenerative therapy outcomes [54]. Tobacco and alcohol use should be formally screened, as both negatively affect MSC function and musculoskeletal regeneration [54]. The SDIMMMER framework provides a structured 7-domain, 35-item assessment tool encompassing Sleep, Diet, Microbiome, Metabolism, Medications, Exams, and Rehabilitation, offering clinicians a systematic approach to metabolic profiling before regenerative therapy [55].

### 6.2. Metabolic Optimization Phase

Based on the evidence reviewed, the following elements may be considered as part of a conceptual, non-prescriptive guide for a 4–8 week optimization phase before orthobiologic therapy, to be individualized at the clinician’s discretion (Table 1): (1) Glycemic optimization with target HbA1c < 8% per ADA perioperative recommendations [140]; (2) Transition to an anti-inflammatory dietary pattern (Mediterranean-style or low DII); (3) Correction of micronutrient deficiencies (vitamin D, magnesium); (4) Adequate protein intake (1.2–1.5 g/kg/day); (5) Consideration of bioactive compound supplementation based on individual patient profiles: berberine (900–1500 mg/day) for AMPK activation, pterostilbene (100–250 mg/day) for SIRT1 activation. Additionally, NAD+ precursors or urolithin A for mitochondrial optimization; and (6) Integration of exercise as tolerated to synergize with nutritional interventions.

For patients with obesity and concurrent osteoarthritis, consideration of GLP-1 receptor agonists (e.g., semaglutide) represents an emerging pharmacological strategy for metabolic optimization before orthobiologic therapy. Bliddal and colleagues demonstrated in a randomized controlled trial that once-weekly semaglutide improved pain, function, and reduced inflammation and bodyweight in patients with knee osteoarthritis and obesity [141]. These benefits may optimize the biological environment for regenerative therapies through anti-inflammatory and weight loss effects, though direct evidence for improved orthobiologic outcomes with GLP-1 receptor agonist pretreatment is not yet available [54].

The proposed 4–8 week optimization window is based on several biological considerations, though direct evidence for optimal timing remains limited (Table 1). First, meaningful changes in glycemic control (reflected in HbA1c) require 2–3 months to manifest, given red blood cell turnover kinetics, supporting intervention initiation at least 8 weeks before therapy. Second, mitochondrial biogenesis adaptations to exercise and nutritional interventions occur over 4–6 weeks based on studies of training adaptations. Third, micronutrient repletion (particularly vitamin D and magnesium) achieves steady-state tissue levels within 4–8 weeks of supplementation initiation. Fourth, dietary pattern changes produce measurable effects on inflammatory markers within 2–4 weeks. For PRP therapy, where the primary goal is optimizing platelet function and the systemic environment, a 4-week minimum may suffice. For BMAC or MSC therapies, where progenitor cell function is paramount, longer optimization periods (8 weeks) may be preferable to allow more complete metabolic reconditioning of the bone marrow niche. These recommendations should be refined based on emerging clinical evidence.

The ESPEN surgical nutrition guidelines provide evidence-based recommendations for perioperative nutritional care that can be adapted for orthobiologic therapy [142]. Prehabilitation principles, including nutritional optimization, exercise, and psychological preparation have demonstrated benefits for surgical outcomes [143,144]. Ljungqvist and colleagues’ review of Enhanced Recovery After Surgery (ERAS) principles provides a framework for integrating nutritional optimization with other perioperative interventions [145].

### 6.3. Peri-Procedural Considerations

Perioperative glycemic management follows established guidelines, with target blood glucose of 100–180 mg/dL per ADA recommendations [140]. The Joint British Diabetes Societies (JBDS) guidelines recommend HbA1c ≤ 8.5% (69 mmol/mol) for elective procedures [146]. Kwon and colleagues demonstrated associations between perioperative hyperglycemia and surgical complications [61].

For PRP preparation specifically, Montagnino et al. suggest that dietary factors may influence platelet function and growth factor release [22]. They recommend avoiding high-fat meals before blood draw, as lipemia can interfere with PRP processing. Adequate hydration facilitates venipuncture and may improve cell yield.

For patients engaged in structured exercise prehabilitation and taking metformin, the potential interaction between metformin and exercise-induced mitochondrial adaptations represents an area of active investigation [99,100]. Continuation of guideline-recommended diabetes pharmacotherapy should not be modified on the basis of current evidence in this context. Clinicians should counsel patients on the importance of maintaining glycemic control throughout prehabilitation and avoid medication changes without robust clinical rationale and appropriate specialist oversight.

### 6.4. Recovery Phase Nutrition

The post-procedure recovery phase (4–12 weeks) requires continued nutritional attention to support tissue regeneration. Key considerations include maintaining adequate protein intake to support collagen synthesis and tissue remodeling; continuing vitamin C supplementation (cofactor for collagen synthesis); ensuring vitamin D adequacy for bone and soft tissue healing; continuing mitochondrial support supplementation; avoiding excessive anti-inflammatory medications that may impair natural healing cascades; and maintaining glycemic control to prevent AGE accumulation in newly synthesized tissue.

The duration of nutritional optimization after orthobiologic therapy should be individualized based on the tissue target (bone healing requires longer support than soft tissue), the specific therapy (stem cell vs. PRP), and patient response. Serial monitoring of inflammatory markers and functional outcomes can guide ongoing nutritional recommendations.

### 6.5. Patient Adherence and Practical Implementation

The comprehensive nature of the optimization protocol raises important questions regarding patient adherence and practical implementation. Multiple dietary changes and supplements over 8–12 weeks represent a significant commitment that may challenge patient compliance.

Strategies to enhance adherence include: (1) prioritizing interventions based on individual patient profiles rather than implementing all recommendations simultaneously; (2) using tiered approaches that begin with dietary modifications and micronutrient correction before adding bioactive compounds; (3) providing structured meal plans and shopping lists aligned with Mediterranean diet principles; (4) utilizing dietitian support for patients requiring intensive intervention; (5) emphasizing food-based approaches over supplements when possible; and (6) setting realistic expectations regarding the rationale for and expected benefits of optimization.

Cost considerations are also relevant, as several recommended supplements (pterostilbene, urolithin A, NAD+ precursors) carry significant expense that may not be covered by insurance. The estimated monthly cost of a comprehensive supplementation protocol may range from $100–300 USD, depending on specific products selected. Clinicians should discuss cost–benefit considerations with patients and prioritize interventions with stronger evidence (vitamin D, magnesium, Mediterranean diet) when resources are limited. The food-based Mediterranean diet approach offers metabolic benefits at relatively modest cost compared to supplement-intensive protocols.

Structured assessment frameworks may facilitate systematic implementation and monitoring of optimization protocols. The SDIMMMER framework proposed by Lana and colleagues provides a quantifiable 7-domain assessment tool that can be used both as a baseline evaluation and as a longitudinal monitoring instrument throughout the optimization period [55]. By systematically tracking progress across Sleep, Diet, Microbiome, Metabolism, Medications, Exams, and Rehabilitation domains, clinicians can identify areas requiring attention, prioritize interventions, and document optimization progress. Integration of such structured tools into clinical workflows may enhance both adherence monitoring and the reproducibility of optimization protocols across different clinical settings.

## 7. Future Directions

### 7.1. Critical Knowledge Gaps

This review identifies several critical knowledge gaps requiring investigation: (1) No randomized trials have directly tested nutritional interventions for improving orthobiologic quality or therapy outcomes in diabetic patients; (2) The NUTRIRISK/REGAIN trial (NCT05546541), examining nutrition status, orthobiologic composition, and clinical outcomes, has completed enrollment but has not yet reported results [147]; (3) Optimal timing and duration of nutritional optimization before orthobiologic therapy remain undefined; (4) Whether autologous therapies can be enhanced through donor optimization, or whether allogeneic sources are preferable for diabetic recipients, requires direct comparison; (5) Biomarkers predicting orthobiologic response in diabetic patients have not been validated; and (6) The relative contributions of mitochondrial biogenesis versus dynamics optimization to regenerative outcomes are unknown; (7) The impact of sleep optimization, microbiome-targeted interventions, and neuroinflammation management on orthobiologic therapy outcomes remains unexplored [54]; (8) Standardized protocols for metabolic screening and optimization in patients undergoing orthobiologic treatments are lacking, and collaborative efforts are needed to develop evidence-based guidelines [54].

### 7.2. Proposed Clinical Studies

We propose the following priority studies to advance this field: First, a randomized trial of structured nutritional prehabilitation (Mediterranean diet, micronutrient optimization, mitochondrial support with berberine/pterostilbene, protein adequacy) versus standard care before PRP therapy for diabetic knee osteoarthritis, with primary outcomes of pain and function at 6 and 12 months. Second, a mechanistic study comparing PRP composition (growth factor profiles, inflammatory mediators, platelet mitochondrial function) before and after 8 weeks of nutritional intervention in T2DM patients. Third, an investigation of AGE accumulation (measured by skin autofluorescence) and mitochondrial biomarkers as predictors of orthobiologic therapy response, potentially enabling patient stratification and targeted intervention.

### 7.3. Methodological Challenges

Rigorous investigation of nutritional optimization for orthobiologic therapy faces several methodological challenges that should inform study design: (1) Standardization of orthobiologic preparations remains problematic, with significant variability in PRP formulations, BMAC processing, and MSC culture methods across centers, necessitating either single-center designs or strict protocol standardization; (2) Heterogeneity of diabetic populations requires careful stratification by disease duration, glycemic control, complications, and medication use; (3) Multiple potential confounding variables (concurrent medications, physical activity, baseline nutritional status) require comprehensive assessment and statistical adjustment; (4) Blinding is challenging for dietary interventions, though feasible for supplement studies; (5) Outcome measures should include both patient-reported outcomes and objective assessments of tissue healing; (6) Sample size calculations must account for the expected variability in orthobiologic responses. Given these complexities, we suggest that initial pilot studies establishing feasibility and preliminary efficacy estimates precede larger definitive trials. Collaboration across multiple centers with expertise in both metabolic medicine and regenerative orthopedics will likely be required.

### 7.4. Biomarker Development

Development of predictive biomarkers for orthobiologic response would enable personalized therapy selection and identify patients most likely to benefit from metabolic optimization. Candidate biomarkers include skin autofluorescence as a measure of tissue AGE accumulation [148,149]; circulating inflammatory markers (IL-6, TNF-α, hsCRP); oxidative stress markers; mitochondrial function assessments (including markers of dynamics such as Mfn1/2, OPA1, and Drp1); and epigenetic signatures of diabetic memory. Machine learning approaches have shown promise for predicting PRP outcomes based on patient characteristics and may incorporate metabolic variables [61]. Integration of metabolic and mitochondrial phenotyping with existing outcome prediction models represents an important research direction.

## 8. Limitations

Several important limitations of this review and the proposed framework should be acknowledged:

First, this is a narrative review rather than a systematic review with formal meta-analytic synthesis. While we have attempted comprehensive coverage of relevant literature, the narrative approach is subject to selection bias and does not provide quantitative effect estimates across studies.

Second, direct evidence connecting nutritional interventions to improved orthobiologic outcomes in diabetic patients is largely absent. The framework proposed herein represents a translational synthesis based on: (a) the established pathophysiology of diabetic regenerative impairment; (b) documented effects of nutritional interventions on relevant molecular pathways; and (c) indirect evidence from related clinical contexts. This evidence pyramid, while mechanistically coherent, requires validation through dedicated clinical trials.

Third, much of the evidence for bioactive compounds derives from preclinical studies or early-phase clinical trials, with limited long-term safety and efficacy data in the specific context of orthobiologic optimization. Extrapolation from mechanistic studies to clinical recommendations involves uncertainty.

Fourth, heterogeneity in both diabetic populations (disease duration, complications, medications, glycemic control) and orthobiologic preparations (PRP formulations, BMAC processing, MSC sources) limits the generalizability of any single intervention protocol. Individualization based on patient characteristics will likely be essential.

Fifth, cost-effectiveness data are absent. The resource implications of comprehensive metabolic optimization, including specialist consultations, laboratory monitoring, and potentially expensive supplements, have not been evaluated against potential benefits.

Sixth, patient-centered outcomes, including quality of life, treatment satisfaction, and preferences for optimization intensity, have not been systematically assessed. The burden of a multi-week prehabilitation protocol may not be acceptable to all patients.

Importantly, several interventions discussed have shown neutral or inconsistent effects in clinical studies, further emphasizing the need for cautious interpretation and context-specific application. These limitations collectively underscore the preliminary nature of current recommendations and the urgent need for rigorous clinical investigation. Seventh, the evidence base is predominantly mechanistic and preclinical: the molecular pathways described in Section 2 derive largely from in vitro studies and rodent models, and the inferential steps required to translate these findings to nutritional interventions in human orthobiologic patients involve substantial uncertainty and a well-recognized risk of translational failure. Eighth, as a narrative review, this work is inherently susceptible to publication bias; studies reporting null or inconsistent effects of nutritional interventions on relevant outcomes may be underrepresented in the available literature. Ninth, the clinical framework proposed in Section 6 is explicitly hypothetical and has not been tested prospectively; synergistic, neutral, or antagonistic interactions between its component interventions remain entirely unknown. The framework should be interpreted as a structured hypothesis warranting prospective evaluation, not as an evidence-based clinical protocol.

## 9. Conclusions

Type 2 diabetes mellitus impairs orthobiologic quality and therapy outcomes through multiple interconnected mechanisms, including AGE accumulation, NF-κB dysregulation, oxidative stress with Nrf2 pathway impairment, mitochondrial dysfunction with impaired biogenesis and dynamics, mesenchymal stem cell dysfunction with diabetic memory, and platelet abnormalities. The tissue microenvironment hypothesis suggests that the recipient tissue milieu, rather than orthobiologic product quality alone, may be the rate-limiting factor for therapy success in diabetic patients.

Nutritional interventions may modulate these pathological mechanisms through effects on inflammatory cascades, mitochondrial function, and metabolic homeostasis, though direct clinical evidence in orthobiologic populations is absent. The most evidence-grounded approaches remain the correction of prevalent micronutrient deficiencies (vitamin D, magnesium) and the adoption of anti-inflammatory dietary patterns such as the Mediterranean diet. Adequate protein intake (1.2–1.5 g/kg/day) is supported by general T2DM nutritional guidelines. Bioactive compounds targeting AMPK, SIRT1, and mitochondrial biogenesis pathways represent mechanistically plausible options warranting dedicated clinical investigation rather than current clinical adoption. For SIRT1 activation, pterostilbene (100–250 mg/day) demonstrates higher bioavailability compared to resveratrol, though its orthobiologic-specific benefit has not been established in clinical trials. For mitochondrial support, NAD+ precursors and urolithin A represent mechanistically plausible but clinically unvalidated options.

We present here a conceptual clinical framework incorporating metabolic assessment 8–12 weeks before orthobiologic therapy, a 4–8-week optimization phase addressing glycemic control, nutritional status, and mitochondrial function, peri-procedural glycemic management, and continued nutritional support during recovery. This framework is a translational synthesis of evidence from diabetic pathophysiology, orthobiologic outcomes, and nutritional science (three domains that have previously remained siloed) and is intended as a conceptual proposal to guide future research and individualized clinical reasoning, not as a prescriptive protocol.

Critical knowledge gaps remain, including the lack of randomized trials directly testing nutritional interventions for orthobiologic optimization in diabetes, undefined optimal timing and duration of interventions, and the absence of validated predictive biomarkers. The limitations of available evidence, including reliance on preclinical data and extrapolation from related clinical contexts, must be acknowledged. Addressing these gaps through rigorous clinical investigation is essential to establish evidence-based protocols for enhancing regenerative medicine outcomes in the growing population of diabetic patients with musculoskeletal pathology.

## Figures and Tables

**Table 1:** Summary of Nutritional Interventions: Level of Evidence and Recommendations.

Intervention	Dose Used in Studies	Level of Evidence *	Primary Mechanism	Key References
Vitamin D	800–2000 IU/day	Moderate (MA)	Bone metabolism, immunity	[84,85,86,87]
Magnesium	200–400 mg/day	Moderate (MA)	Insulin sensitivity, bone health	[89,90]
Protein intake	1.2–1.5 g/kg/day	Moderate (GL)	Tissue repair, sarcopenia prevention	[68,69,70]
Omega-3 FA	2–4 g/day	Moderate (RCT)	Anti-inflammatory, SPM generation	[82,83]
Berberine	900–1500 mg/day	Moderate (MA)	AMPK activation, glucose control	[93,94,95,96]
Pterostilbene	100–250 mg/day	Low (Preclinical)	SIRT1 activation	[97,98,99,100]
NAD+ precursors	250–1000 mg/day	Low (Early RCT)	Sirtuin activation, MITO function	[101]
Urolithin A	500–1000 mg/day	Low (Early RCT)	Mitophagy, MITO biogenesis	[102,103]
Sulforaphane	Dietary sources	Moderate (Preclinical)	Nrf2 activation	[33,104]
Mediterranean diet	Pattern-based	High (MA)	Anti-inflammatory, metabolic	[105,106,107,108]
Low-AGE diet	Cooking methods	Moderate (MA)	AGE burden reduction	[75,79,80]

* Level of evidence: High = multiple large RCTs or meta-analyses (MA); Moderate = smaller RCTs, meta-analyses with limitations, or strong guideline (GL) support; Low = primarily preclinical data, early-phase RCTs, or mechanistic studies. All recommendations should be individualized based on patient characteristics and potential interactions. The level of evidence reflects the highest level of available evidence for each intervention and is intended to guide interpretation of the data discussed throughout the manuscript. Doses reflect ranges reported in the literature and should not be interpreted as clinical recommendations.

## Data Availability

No new data were created or analyzed in this study. Data sharing is not applicable to this article.

## Abbreviations

The following abbreviations are used in this manuscript:

AGEs	Advanced glycation end-products
AMPK	AMP-activated protein kinase
ARE	Antioxidant response element
BMAC	Bone marrow aspirate concentrate
CFU	Colony-forming units
CoQ10	Coenzyme Q10
CR	Caloric restriction
DII	Dietary inflammatory index
Drp1	Dynamin-related protein 1
EPA/DHA	Eicosapentaenoic acid/Docosahexaenoic acid
ETC	Electron transport chain
FMD	Fasting-mimicking diet
GLIM	Global Leadership Initiative on Malnutrition
HbA1c	Glycated hemoglobin
hsCRP	High-sensitivity C-reactive protein
IF	Intermittent fasting
Keap1	Kelch-like ECH-associated protein 1
MAPK	Mitogen-activated protein kinase
Mfn1/2	Mitofusin 1/2
MSC	Mesenchymal stem/stromal cell
mTOR	Mechanistic target of rapamycin
NAD+	Nicotinamide adenine dinucleotide
NF-κB	Nuclear factor kappa-light-chain-enhancer of activated B cells
NMN	Nicotinamide mononucleotide
NR	Nicotinamide riboside
Nrf2	Nuclear factor erythroid 2-related factor 2
NRS-2002	Nutritional Risk Screening 2002
OA	Osteoarthritis
OPA1	Optic atrophy 1
PGC-1α	Peroxisome proliferator-activated receptor gamma coactivator 1-alpha
PINK1	PTEN-induced kinase 1
PRP	Platelet-rich plasma
RAGE	Receptor for advanced glycation end-products
RCT	Randomized controlled trial
ROS	Reactive oxygen species
SAF	Skin autofluorescence
SIRT1/3	Sirtuin 1/3
SPM	Specialized pro-resolving mediators
T2DM	Type 2 diabetes mellitus
TRF	Time-restricted feeding
VAS	Visual analog scale
WOMAC	Western Ontario and McMaster Universities Osteoarthritis Index
β-HB	β-hydroxybutyrate

## References

[1] Sun H., Saeedi P., Karuranga S., Pinkepank M., Ogurtsova K., Duncan B.B., Stein C., Basit A., Chan J.C.N., Mbanya J.C. (2022). IDF Diabetes Atlas: Global, regional and country-level diabetes prevalence estimates for 2021 and projections for 2045. Diabetes Res. Clin. Pract..

[2] Wukich D.K., Crim B.E., Frykberg R.G., Rosario B.L. (2014). Neuropathy and poorly controlled diabetes increase the rate of surgical site infection after foot and ankle surgery. J. Bone Joint. Surg. Am..

[3] Diaz R., DeJesus J. (2021). Managing Patients Undergoing Orthopedic Surgery to Improve Glycemic Outcomes. Curr. Diabetes Rep..

[4] Wukich D.K. (2015). Diabetes and its negative impact on outcomes in orthopaedic surgery. World J. Orthop..

[5] Ding Z.C., Zeng W.N., Rong X., Liang Z.M., Zhou Z.K. (2020). Do patients with diabetes have an increased risk of impaired fracture healing? A systematic review and meta-analysis. ANZ J. Surg..

[6] Lui P.P.Y. (2017). Tendinopathy in diabetes mellitus patients-Epidemiology, pathogenesis, and management. Scand. J. Med. Sci. Sports.

[7] Best S.L., Rios F.M., Gongora L.L., Haynes E.C., Knoop R.L. (2019). The Effects of Type II Diabetes Mellitus on Tendon Homeostasis and Healing. J. Diabetes Res..

[8] Stausholm M.B., Kristensen M.T., Rahbek O., Overgaard S., Pedersen A.B. (2024). Increased Risk of Tendon Injury Following Structured Care in Patients with Type 2 Diabetes: Post Hoc Analysis of a Large Randomized Controlled Trial with 19 Years of Follow-up. Diabetes Care.

[9] Papadogeorgis P., Karaliotas A., Korakakis V., Kotsifaki R., Zacharouli E., Roumpi T. (2025). Platelet-Rich Plasma for Knee Osteoarthritis: A Comprehensive Narrative Review. J. Clin. Med..

[10] Go E.J., Kang J.H., Kim J. (2025). Bone Marrow Aspirate Concentrate (BMAC) for Knee Osteoarthritis: A Narrative Review of Clinical Efficacy and Future Directions. Medicina.

[11] Adams S.B., Leimer E.M., Setton L.A., Nettles D.L., Olson S.A., Gilmore C.J. (2025). Advancements in Regenerative Therapies for Orthopedics: A Comprehensive Review. Int. J. Mol. Sci..

[12] Park J.S., Park H.J., Kim S.H., Oh J.H. (2020). Clinical and Structural Outcomes After Rotator Cuff Repair in Patients with Diabetes: A Meta-analysis. Am. J. Sports Med..

[13] Abate M., Di Carlo L., Salini V., Schiavone C. (2024). Platelet Rich Plasma Therapy in Achilles and Patellar Tendinopathies: Outcomes in Subjects with Diabetes. J. Clin. Med..

[14] Chang T.C., Hsu M.F., Wu K.K. (2015). High glucose induces bone marrow-derived mesenchymal stem cell senescence by upregulating autophagy. PLoS ONE.

[15] He X., Wang H., Jin T., Xu Y., Mei L., Yang J. (2023). Osteogenesis of bone marrow mesenchymal stem cell in hyperglycemia. Front. Endocrinol..

[16] Suzuki A., Yabu A., Nakamura H. (2022). Advanced glycation end products in musculoskeletal system and disorders. Methods.

[17] Patel S.H., Yue F., Garg K., Huang S., Shin J.Y., Shah P.K. (2019). Advanced Glycation End-Products Suppress Mitochondrial Function and Proliferative Capacity of Achilles Tendon-Derived Fibroblasts. Sci. Rep..

[18] Giacco F., Brownlee M. (2010). Oxidative stress and diabetic complications. Circ. Res..

[19] Natarajan R. (2021). Epigenetic Mechanisms in Diabetic Vascular Complications and Metabolic Memory: The 2020 Edwin Bierman Award Lecture. Diabetes.

[20] Patti M.E., Butte A.J., Crunkhorn S., Cusi K., Berria R., Kashyap S., Miyazaki Y., Kohane I., Costello M., Saccone R. (2003). Coordinated reduction of genes of oxidative metabolism in humans with insulin resistance and diabetes: Potential role of PGC1 and NRF1. Proc. Natl. Acad. Sci. USA.

[21] Bhansali S., Bhansali A., Walia R., Saikia U.N., Dhawan V. (2017). Alterations in Mitochondrial Oxidative Stress and Mitophagy in Subjects with Prediabetes and Type 2 Diabetes Mellitus. Front. Endocrinol..

[22] Montagnino J., Kaufman M.W., Shetty M., Centeno C., Fredericson M. (2025). Optimizing orthobiologic therapies with exercise, diet, and supplements. PM&R.

[23] Everts P.A., van Erp A., DeSimone A., Cohen D.S., Gardner R.D. (2021). Platelet Rich Plasma in Orthopedic Surgical Medicine. Platelets.

[24] Saito M., Kida Y., Kato S., Marumo K. (2014). Diabetes, Collagen, and Bone Quality. Curr. Osteoporos. Rep..

[25] Zhou Z., Immel D., Xi C.X., Bhattacharyya R.S., Bhatti S.K. (2006). Regulation of osteoclast function and bone mass by RAGE. J. Exp. Med..

[26] Davis H.M., Essex A.L., Valdez S., Deosthale P.J., Aref M.W., Allen M.R., Bonetto A., Plotkin L.I. (2019). Short-term pharmacologic RAGE inhibition differentially affects bone and skeletal muscle in middle-aged mice. Bone.

[27] Taguchi K., Fukami K. (2023). RAGE signaling regulates the progression of diabetic complications. Front. Pharmacol..

[28] Saito M., Marumo K. (2013). Bone quality in diabetes. Front. Endocrinol..

[29] Ko K.I., Coimbra L.S., Tian C., Alber J., Enber M.D., Kang P., Abraham T.W., Bhattacharyya A., Bhutani S., Graves D.T. (2019). Diabetes-Induced NF-κB Dysregulation in Skeletal Stem Cells Prevents Resolution of Inflammation. Diabetes.

[30] Baker R.G., Hayden M.S., Ghosh S. (2011). NF-κB, inflammation, and metabolic disease. Cell Metab..

[31] Long M., Rojo de la Vega M., Wen Q., Bharara M., Lee S.S., Coursen J.D., Wang X., Roop D.R., Armstrong D.G., Zhang D.D. (2016). An Essential Role of NRF2 in Diabetic Wound Healing. Diabetes.

[32] Soares M.A., Cohen O.D., Low Y.C., Sarber R.A., Anahari T., Galiano R.D. (2016). Restoration of Nrf2 Signaling Normalizes the Regenerative Niche. Diabetes.

[33] Soares M.A., Ezeamuzie O.C., Ham M.J., Duckworth A.M., Rabbani P.S., Saadeh P.B. (2019). Targeted Protection of Donor Graft Vasculature Using a Nrf2 Stimulator Confers Long-lasting Allograft Survival. Diabetes.

[34] Dodson M., Shakya A., Anandhan A., Chen J., Garcia J.G.N., Zhang D.D. (2022). NRF2 and Diabetes: The Good, the Bad, and the Complex. Diabetes.

[35] David J.A., Bhimji S.S. (2023). The Nrf2 Signaling Pathway in Diabetes. StatPearls.

[36] Kelley D.E., He J., Menshikova E.V., Ritov V.B. (2002). Dysfunction of mitochondria in human skeletal muscle in type 2 diabetes. Diabetes.

[37] Patti M.E., Corvera S. (2010). The role of mitochondria in the pathogenesis of type 2 diabetes. Endocr. Rev..

[38] Shares B.H., Busch M., White N., Shum L., Elber M.D. (2018). Active mitochondria support osteogenic differentiation by stimulating β-catenin acetylation. J. Biol. Chem..

[39] Youle R.J., van der Bliek A.M. (2012). Mitochondrial fission, fusion, and stress. Science.

[40] Phinney D.G., Di Giuseppe M., Njah J., Sala E., Shiva S., St Croix C.M., Stolz D.B., Watkins S.C., Di Y.P., Bhattacharyya A. (2015). Mesenchymal stem cells use extracellular vesicles to outsource mitophagy and shuttle microRNAs. Nat. Commun..

[41] Islam M.N., Das S.R., Bhattacharyya A., Bhattacharyya J. (2012). Transfer of mitochondria from bone marrow-derived stromal cells to pulmonary alveoli protects against acute lung injury. Nat. Med..

[42] Reddy M.A., Zhang E., Natarajan R. (2015). Epigenetic mechanisms in diabetic complications and metabolic memory. Diabetologia.

[43] Rennert R.C., Sorkin M., Januszyk M., Duscher D., Kosaraju R., Chung M.T., Lennon J., Radber A., Longaker M.T., Gurtner G.C. (2014). Diabetes impairs the angiogenic potential of adipose-derived stem cells by selectively depleting cellular subpopulations. Stem Cell Res. Ther..

[44] Yu J., Shi J., Zhang Y., Zhang Y., Huang Y., Chen Z., Yang J. (2022). Stem Cell-Based Therapy: A Promising Treatment for Diabetic Foot Ulcer. Front. Bioeng. Biotechnol..

[45] Vinik A.I., Erbas T., Park T.S., Nolan R., Pittenger G.L. (2001). Platelet dysfunction in type 2 diabetes. Diabetes Care.

[46] Karina K., Rosliana I., Sobariah S., Rosadi I., Widyastuti T., Afini I., Rosyadi I., Barlian A., Soewondo P., Pawitan J.A. (2019). Evaluation of platelet-rich plasma from diabetic donors shows increased platelet vascular endothelial growth factor release. Stem Cell Investig..

[47] Kakouros N., Rade J.J., Kourliouros A., Resar J.R. (2011). Platelet function in patients with diabetes mellitus: From a theoretical to a practical perspective. Int. J. Endocrinol..

[48] Shahid S., Khan Z.A. (2018). Platelets and Cardiovascular Disease in Diabetes. Cardiovasc. Diabetol..

[49] Li X., Wu G., Han F., Wang K., Bai X., Jia Y., Li Z., Cai W., Zhang W., Su L. (2019). SIRT1 activation promotes angiogenesis in diabetic wounds by protecting endothelial cells against oxidative stress. Arch. Biochem. Biophys..

[50] Berlanga-Acosta J.A., Guillén-Nieto G.E., Rodríguez-Rodríguez N., Mendoza-Marí Y., Bringas-Vega M.L., Berlanga-Saez J.O., García Del Barco Herrera D., Martinez-Jimenez I., Hernandez-Gutierrez S., Valdés-Sosa P.A. (2020). Cellular Senescence as the Pathogenic Hub of Diabetes-Related Wound Chronicity. Front. Endocrinol..

[51] Wang Y., Graves D.T. (2022). Macrophage plasticity and inflammation in diabetic wound healing. Burns Trauma.

[52] Al-Mulla F., Leibovich S.J., Francis I.M., Bitar M.S. (2011). Impaired TGF-β signaling and a defect in resolution of inflammation contribute to delayed wound healing in a female rat model of type 2 diabetes. Mol. Biosyst..

[53] Dong H., Sun Y., Nie L., Cui A., Zhao P., Leung W.K., Wang Q. (2024). Epigenetic regulation of macrophage polarization in diabetic wound healing. Mol. Med. Rep..

[54] Fernandes G.C.A.M., Rodeo S.A. (2026). Metabolic Optimization Before Orthobiologic Therapies (MOBOT): A Narrative Review. Sports Health.

[55] Lana J.V., Lana J.F., Melo G., Azzini G.O.M., Santos G.S., Mosaner T., Jorge D.d.M.F., da Fonseca L.F., Kruel A., Costa F.R. (2024). SDIMMMER: A Proposed Clinical Approach to Optimize Cellular Physiology in Regenerative Medicine. Life.

[56] Xu H., Huang K., Tao X., Huang W., Huang M. (2025). Efficacy and safety of platelet-rich plasma versus conventional care in diabetic foot ulcers: A meta-analysis of randomized controlled trials. Acta Diabetol..

[57] Meznerics F.A., Fehérvári P., Dembrovszky F., Kovács K.D., Kemény L.V., Csupor D., Hegyi P., Bánvölgyi A. (2022). Platelet-Rich Plasma in Chronic Wound Management: A Systematic Review and Meta-Analysis. J. Clin. Med..

[58] Su Y.N., Zhao D.Y., Li Y.H., Gao Z.H., Yu J.J. (2023). Platelet-rich plasma for diabetic foot ulcer: A systematic review and meta-analysis. J. Orthop. Surg. Res..

[59] Martinez-Zapata M.J., Martí-Carvajal A.J., Solà I., Expósito J.A., Bolíbar I., Rodríguez L., Garcia J., Zaror C. (2016). Autologous platelet-rich plasma for treating chronic wounds. Cochrane Database Syst. Rev..

[60] Del Pino-Sedeño T., Trujillo-Martín M.M., Andia I., Aragón-Sánchez J., Herrera-Ramos E., Iruzubieta Barragán F.J., Serrano-Aguilar P. (2019). Platelet-rich plasma for the treatment of diabetic foot ulcers: A meta-analysis. Wound Repair Regen..

[61] Kwon S., Thompson R., Dellinger P., Yanez D., Farrohki E., Flum D. (2013). Importance of perioperative glycemic control in general surgery: A report from the Surgical Care and Outcomes Assessment Program. Ann. Surg..

[62] Kashbour M., Alem M.M., Ghanem K., Elshazly M.B. (2025). Mesenchymal stem cell-based therapy for type 1 & 2 diabetes mellitus patients: A systematic review and meta-analysis of randomized controlled trials. Diabetol. Metab. Syndr..

[63] Wang L., Zhao Y., Shi S. (2021). Efficacy of mesenchymal stem cell transplantation therapy for type 1 and type 2 diabetes mellitus: A meta-analysis. Stem Cell Res. Ther..

[64] Tong L., Chen W., Wu R., Wu Y., Liu Y., Zhong Z., Sun Y. (2025). Impacts of stem cells from different sources on wound healing rate in diabetic foot ulcers: A systematic review and meta-analysis. Front. Genet..

[65] Çelik D., Eryigit Ü., Simsek H.H., Yildiz Ö., Akdeniz H., Özdemir E., Coskun F. (2023). The impact of diabetes mellitus on hematopoietic stem cell mobilization. Transfus. Apher. Sci..

[66] American Diabetes Association Professional Practice Committee (2025). Standards of Care in Diabetes-2025. Diabetes Care.

[67] (2023). Diabetes and Nutrition Study Group (DNSG) of the European Association for the Study of Diabetes (EASD). Evidence-based European recommendations for the dietary management of diabetes. Diabetologia.

[68] Feng L., Gao Q., Hu K., Wu M., Wang Z., Chen F., Mei F., Zhao L., Ma B. (2023). Pathogenesis and comprehensive treatment strategies of sarcopenia in elderly patients with type 2 diabetes mellitus. Front. Endocrinol..

[69] Dodd K.M., Tee A.R. (2012). Leucine and mTORC1: A complex relationship. Am. J. Physiol. Endocrinol. Metab..

[70] Liu Y., Chen X., Zhang Y., Wang J. (2023). Research progress in the role and mechanism of Leucine in regulating animal growth and development. Front. Physiol..

[71] Leenders M., Verdijk L.B., van der Hoeven L., Adam J.J., van Kranenburg J., Kuipers H., van Loon L.J. (2013). Protein supplementation during resistance-type exercise training in the elderly. Med. Sci. Sports Exerc..

[72] Fusaro M., Cianciolo G., Evenepoel P., Abaterusso C., Plebani M., Zaninotto M., Giannini S. (2022). The impact of nutrition on tendon health and tendinopathy: A systematic review. J. Int. Soc. Sports Nutr..

[73] Baye E., Kiriakova V., Uribarri J., Moran L.J., de Courten B. (2017). Consumption of diets with low advanced glycation end products improves cardiometabolic parameters: Meta-analysis of randomised controlled trials. Sci. Rep..

[74] de Courten B., de Courten M.P., Soldatos G., Dougherty S.L., Straznicky N., Schlaich M., Sourris K.C., Chand V., Tziomalos K., Kingwell B.A. (2016). Diet low in advanced glycation end products increases insulin sensitivity in healthy overweight individuals: A double-blind, randomized, crossover trial. Am. J. Clin. Nutr..

[75] Mark A.B., Poulsen M.W., Andersen S., Andersen J.M., Bak M.J., Ritz C., Holst J.J., Nielsen J., de Courten B., Dragsted L.O. (2014). Consumption of a diet low in advanced glycation end products for 4 weeks improves insulin sensitivity in overweight women. Diabetes Care.

[76] Shukla A.P., Dickison M., Coughlin N., Karan A., Mauer E., Truong W., Casper A., Igel L.I., Kumar R.B., Saunders K.H. (2017). Carbohydrate-Last Meal Pattern Lowers Postprandial Glucose and Insulin Excursions in Type 2 Diabetes. BMJ Open Diabetes Res. Care.

[77] Li Y., Wang X. (2022). Chrysin Attenuates High Glucose-Induced Bone Marrow Stromal Cell Apoptosis and Dysfunction via the Activation of the PI3K/AKT/Nrf2 Signaling Pathway. Drug Des. Devel. Ther..

[78] Ma J., Stevens J.E., Cukier K., Maddox A.F., Wishart J.M., Jones K.L., Clifton P.M., Horowitz M., Rayner C.K. (2009). Effects of a Protein Preload on Gastric Emptying, Glycemia, and Gut Hormones After a Carbohydrate Meal in Diet-Controlled Type 2 Diabetes. Diabetes Care.

[79] Serini S., Calviello G. (2021). Omega-3 Fatty Acids and Their Anti-Inflammatory Role in the Prevention and Treatment of Diabetic Nephropathy. Foods.

[80] Soleimani A., Mojarrad M.Z., Bahmani F., Taghizadeh M., Ramezani M., Tajabadi-Ebrahimi M., Jafari P., Esmaillzadeh A., Asemi Z. (2024). Effect of flaxseed oil supplementation on diabetic foot ulcer. Appl. Sci..

[81] Sandford F.M., Sanders T.A.B., Wilson H., Lewis J.S. (2018). A randomised controlled trial of long-chain omega-3 polyunsaturated fatty acids in the management of rotator cuff related shoulder pain. BMJ Open Sport Exerc. Med..

[82] Llombart A., Marques L., Riquelme F., Bea A.M., Balcells E., Ortega A., Cuesta F., Mateo L. (2024). Impact of vitamin D deficiency on mortality in patients with hip fracture: A meta-analysis. J. Am. Geriatr. Soc..

[83] Kong S.H., Jang H.N., Kim J.H., Kim S.W., Shin C.S. (2022). Effect of Vitamin D Supplementation on Risk of Fractures and Falls According to Dosage and Interval: A Meta-Analysis. Endocrinol. Metab..

[84] Holick M.F. (2007). Vitamin D deficiency. N. Engl. J. Med..

[85] LeBoff M.S., Chou S.H., Ratliff K.A., Cook N.R., Khurana B., Kim E., Cawthon P.M., Bauer D.C., Black D., Gallagher J.C. (2022). Supplemental Vitamin D and Incident Fractures in Midlife and Older Adults. N. Engl. J. Med..

[86] Xu L., Li X., Wang X., Xu M. (2023). Effects of magnesium supplementation on improving hyperglycemia, hypercholesterolemia, and hypertension in type 2 diabetes: A pooled analysis of 24 randomized controlled trials. Front. Nutr..

[87] Simental-Mendía L.E., Sahebkar A., Rodríguez-Morán M., Guerrero-Romero F. (2016). A systematic review and meta-analysis of randomized controlled trials on the effects of magnesium supplementation on insulin sensitivity and glucose control. Pharmacol. Res..

[88] Farsinejad-Marj M., Saneei P., Esmaillzadeh A. (2016). Dietary magnesium intake, bone mineral density and risk of fracture: A systematic review and meta-analysis. Osteoporos. Int..

[89] Moore Z.E., Corcoran M.A., Patton D. (2020). Nutritional interventions for treating foot ulcers in people with diabetes. Cochrane Database Syst. Rev..

[90] Lin P.H., Sermersheim M., Li H., Lee P.H.U., Steinberg S.M., Ma J. (2018). Zinc in Wound Healing Modulation. Nutrients.

[91] DePhillipo N.N., Aman Z.S., Kennedy M.I., Begley J.P., Moatshe G., LaPrade R.F. (2018). Efficacy of Vitamin C Supplementation on Collagen Synthesis and Oxidative Stress After Musculoskeletal Injuries: A Systematic Review. Orthop. J. Sports Med..

[92] Shaw G., Lee-Barthel A., Ross M.L., Wang B., Baar K. (2017). Vitamin C-enriched gelatin supplementation before intermittent activity augments collagen synthesis. Am. J. Clin. Nutr..

[93] Lan J., Zhao Y., Dong F., Yan Z., Zheng W., Fan J., Sun G. (2015). Meta-analysis of the effect and safety of berberine in the treatment of type 2 diabetes mellitus, hyperlipemia and hypertension. J. Ethnopharmacol..

[94] Shang W., Liu J., Yu X., Zhao J. (2010). Effects of berberine on serum levels of inflammatory factors and inflammatory signaling pathway in obese mice induced by high fat diet. Zhongguo Zhong Yao Za Zhi.

[95] Senger D.R., Li D., Jaminet S.C., Cao S. (2016). Activation of the Nrf2 Cell Defense Pathway by Ancient Foods: Disease Prevention by Important Molecules and Microbes Lost from the Modern Western Diet. PLoS ONE.

[96] Cuadrado A., Rojo A.I., Wells G., Hayes J.D., Cousin S.P., Rumsey W.L., Attucks O.C., Franklin S., Levonen A.L., Kensler T.W. (2019). Therapeutic targeting of the NRF2 and KEAP1 partnership in chronic diseases. Nat. Rev. Drug Discov..

[97] Huang X., Sun J., Chen G., Niu C., Wang Y., Zhao C., Sun J., Huang H., Huang S., Liang Y. (2019). Resveratrol Promotes Diabetic Wound Healing via SIRT1-FOXO1-c-Myc Signaling Pathway-Mediated Angiogenesis. Front. Pharmacol..

[98] Hardie D.G. (2013). AMPK: A target for drugs and natural products with effects on both diabetes and cancer. Diabetes.

[99] Walton R.G., Dungan C.M., Long D.E., Tuggle S.C., Kosmac K., Ponce González J.G., Kern P.A., Rasmussen B.B., Peterson C.A. (2019). Metformin blunts muscle hypertrophy in response to progressive resistance exercise training in older adults: A randomized, double-blind, placebo-controlled, multicenter trial: The MASTERS trial. Aging Cell.

[100] Konopka A.R., Laurin J.L., Schoenberg H.M., Reid J.J., Castor W.M., Wolff C.A., Musci R.V., Safdar A., Miller B.F., Hamilton K.L. (2019). Metformin inhibits mitochondrial adaptations to aerobic exercise training in older adults. Aging Cell.

[101] Littarru G.P., Langsjoen P. (2007). Coenzyme Q10 and statins: Biochemical and clinical implications. Mitochondrion.

[102] Ziegler D., Hanefeld M., Ruhnau K.J., Meissner H.P., Lobisch M., Schütte K., Gries F.A. (1995). Treatment of symptomatic diabetic peripheral neuropathy with the anti-oxidant alpha-lipoic acid. A 3-week multicentre randomized controlled trial (ALADIN Study). Diabetologia.

[103] Ziegler D., Low P.A., Litchy W.J., Boulton A.J., Vinik A.I., Freeman R., Samigullin R., Tritschler H., Munzel U., Maus J. (2011). Efficacy and safety of antioxidant treatment with α-lipoic acid over 4 years in diabetic polyneuropathy: The NATHAN 1 trial. Diabetes Care.

[104] Andreux P.A., Blanco-Bose W., Ryu D., Burber F., Park J.S., Aebischer P., Auwerx J., Singh A., Rinsch C. (2019). The mitophagy activator urolithin A is safe and induces a molecular signature of improved mitochondrial and cellular health in humans. Nat. Metab..

[105] Schwingshackl L., Chaimani A., Hoffmann G., Schwedhelm C., Boeing H. (2018). A network meta-analysis on the comparative efficacy of different dietary approaches on glycaemic control in patients with type 2 diabetes mellitus. Eur. J. Epidemiol..

[106] Cerletti M., Jang Y.C., Finley L.W., Haigis M.C., Wagers A.J. (2012). Short-term calorie restriction enhances skeletal muscle stem cell function. Cell Stem Cell.

[107] Maharajan N., Cho G.W., Jang C.H. (2020). Caloric restriction maintains stem cells through niche and cell modulation. J. Mol. Med..

[108] Mihaylova M.M., Cheng C.W., Cao A.Q., Tripathi S., Mana M.D., Bauer-Rowe K.E., Abu-Remaileh M., Clavain L., Erber A., Balanis N.G. (2018). Fasting Activates Fatty Acid Oxidation to Enhance Intestinal Stem Cell Function during Homeostasis and Aging. Cell Stem Cell.

[109] Milne J.C., Lambert P.D., Schenk S., Carney D.P., Smith J.J., Gagne D.J., Jin L., Boss O., Perni R.B., Vu C.B. (2007). Small molecule activators of SIRT1 as therapeutics for the treatment of type 2 diabetes. Nature.

[110] Kapetanovic I.M., Muzzio M., Huang Z., Thompson T.N., McCormick D.L. (2011). Pharmacokinetics, oral bioavailability, and metabolic profile of resveratrol and its dimethylether analog, pterostilbene, in rats. Cancer Chemother. Pharmacol..

[111] Riche D.M., McEwen C.L., Riche K.D., Sherman J.J., Wofford M.R., Deschamp D., Griswold M. (2013). Analysis of safety from a human clinical trial with pterostilbene. J. Toxicol..

[112] McCormack D., McFadden D. (2013). A review of pterostilbene antioxidant activity and disease modification. Oxid. Med. Cell. Longev..

[113] Yin J., Xing H., Ye J. (2008). Efficacy of berberine in patients with type 2 diabetes mellitus. Metabolism.

[114] Zhang H., Wei J., Xue R., Wu J.D., Zhao W., Wang Z.Z., Wang S.K., Zhou Z.X., Song D.Q., Wang Y.M. (2010). Berberine lowers blood glucose in type 2 diabetes mellitus patients through increasing insulin receptor expression. Metabolism.

[115] Dong H., Wang N., Zhao L., Lu F. (2012). Berberine in the treatment of type 2 diabetes mellitus: A systemic review and meta-analysis. Evid. Based Complement. Alternat. Med..

[116] Elhassan Y.S., Kluckova K., Fletcher R.S., Schmidt M.S., Garten A., Doig C.L., Cartwright D.M., Oakey L., Burley C.V., Jenkinson N. (2019). Nicotinamide riboside augments the aged human skeletal muscle NAD+ metabolome and induces transcriptomic and anti-inflammatory signatures. Cell Rep..

[117] Yoshino M., Yoshino J., Kayser B.D., Patti G.J., Franczyk M.P., Mills K.F., Sindelar M., Pietka T., Patterson B.W., Imai S.I. (2021). Nicotinamide mononucleotide increases muscle insulin sensitivity in prediabetic women. Science.

[118] Liu S., D’Amico D., Shankland E., Bhardwaj G., Morton D.J., Bhutani S., Parise R., Singh A., Rinsch C., Auwerx J. (2022). Effect of urolithin A supplementation on muscle endurance and mitochondrial health in older adults: A randomized clinical trial. JAMA Netw. Open.

[119] Agathos E., Tentolouris A., Eleftheriadou I., Katsaouni P., Nemtzas I., Petrou A., Papanikolaou C., Tentolouris N. (2018). Effect of α-lipoic acid on symptoms and quality of life in patients with painful diabetic neuropathy. J. Int. Med. Res..

[120] Esposito K., Maiorino M.I., Bellastella G., Chiodini P., Panagiotakos D., Giugliano D. (2015). A journey into a Mediterranean diet and type 2 diabetes: A systematic review with meta-analyses. BMJ Open.

[121] Estruch R., Ros E., Salas-Salvadó J., Covas M.I., Corella D., Arós F., Gómez-Gracia E., Ruiz-Gutiérrez V., Fiol M., Lapetra J. (2018). Primary Prevention of Cardiovascular Disease with a Mediterranean Diet Supplemented with Extra-Virgin Olive Oil or Nuts. N. Engl. J. Med..

[122] Papadaki A., Nolen-Doerr E., Mantzoros C.S. (2020). The Effect of the Mediterranean Diet on Metabolic Health: A Systematic Review and Meta-Analysis of Controlled Trials in Adults. Nutrients.

[123] Imada S., Khawaled S., Shin H., Meckelmann S.W., Greer C., Tanaka T., Thakker N., Singh S., Banerjee P., Nguyen D.N. (2024). Post-fast refeeding enhances intestinal stem cell-mediated epithelial regeneration and tumourigenesis. Nature.

[124] Choi Y.J., Jeon S.M., Shin S. (2020). Impact of a Ketogenic Diet on Metabolic Parameters in Patients with Obesity or Overweight and with or without Type 2 Diabetes: A Meta-Analysis of Randomized Controlled Trials. Nutrients.

[125] Choy K.Y.C., Louie J.C.Y. (2023). The effects of the ketogenic diet for the management of type 2 diabetes mellitus: A systematic review and meta-analysis of recent studies. Diabetes Metab. Syndr..

[126] Parry-Strong A., Wright-McNaughton M., Weatherall M., Hall R.M., Coppell K.J., Barthow C., Krebs J.D. (2022). Very low carbohydrate (ketogenic) diets in type 2 diabetes: A systematic review and meta-analysis of randomized controlled trials. Diabetes Obes. Metab..

[127] Shimazu T., Hirschey M.D., Newman J., He W., Shirakawa K., Le Moan N., Grueter C.A., Lim H., Saunders L.R., Stevens R.D. (2013). Suppression of oxidative stress by β-hydroxybutyrate, an endogenous histone deacetylase inhibitor. Science.

[128] Newman J.C., Verdin E. (2017). β-Hydroxybutyrate: A Signaling Metabolite. Annu. Rev. Nutr..

[129] Puchalska P., Crawford P.A. (2021). Metabolic and Signaling Roles of Ketone Bodies in Health and Disease. Annu. Rev. Nutr..

[130] Han Y.M., Bedarida T., Ding Y., Somber B.K., Lu Q., Wang Q., Song P., Zou M.H. (2018). β-Hydroxybutyrate Prevents Vascular Senescence through hnRNP A1-Mediated Upregulation of Oct4. Mol. Cell.

[131] Shivappa N., Steck S.E., Hurley T.G., Hussey J.R., Hébert J.R. (2014). Designing and developing a literature-derived, population-based dietary inflammatory index. Public Health Nutr..

[132] Li J., Lee D.H., Hu J., Tabung F.K., Li Y., Bhupathiraju S.N., Rimm E.B., Rexrode K.M., Manson J.E., Willett W.C. (2022). Dietary Inflammatory Potential and Risk of Type 2 Diabetes: A Prospective Study of 51,529 Adults. Clin. Nutr..

[133] Hariharan R., Odjidja E.N., Scott D., Shivappa N., Hébert J.R., Hodge A., de Courten B. (2022). The dietary inflammatory index, obesity, type 2 diabetes, and cardiovascular risk factors and diseases. Obes. Rev..

[134] Wang Q., Zhang J., Liang Z., Li Y., Liu X. (2022). Association between dietary inflammatory index and type 2 diabetes: NHANES 2005–2018. Front. Endocrinol..

[135] Ozawa M., Shipley M., Kivimaki M., Singh-Manoux A., Brunner E.J. (2022). Dietary inflammatory index and long-term changes in body composition: The Whitehall II study. Eur. J. Clin. Nutr..

[136] Sun Z., Kong X.J., Jing X., Deng R.J., Tian Z.B. (2015). Nutritional Risk Screening 2002 as a Predictor of Postoperative Outcomes in Patients Undergoing Abdominal Surgery: A Systematic Review and Meta-Analysis. PLoS ONE.

[137] Ozkalkanli M.Y., Ozkalkanli D.T., Katircioglu K., Savaci S. (2009). Comparison of Tools for Nutrition Assessment and Screening for Predicting the Development of Complications in Orthopedic Surgery. Nutr. Clin. Pract..

[138] Cheung H.H.T., Pufulete M., Ciardullo E., Shea R. (2024). Comparison of nutritional screening and assessment tools: A network meta-analysis. Int. J. Surg..

[139] Cederholm T., Jensen G.L., Correia M.I.T.D., Gonzalez M.C., Fukushima R., Higashiguchi T., Baptista G., Barazzoni R., Blaauw R., Coats A. (2019). GLIM criteria for the diagnosis of malnutrition—A consensus report from the global clinical nutrition community. J. Cachexia Sarcopenia Muscle.

[140] American Diabetes Association Professional Practice Committee (2025). 16. Diabetes Care in the Hospital: Standards of Care in Diabetes-2025. Diabetes Care.

[141] Bliddal H., Bays H., Czernichow S., Hemmingsson J.U., Hjelmesæth J., Morville T.H., Koroleva A., Neergaard J.S., Sánchez P.V., Wharton S. (2024). Once-Weekly Semaglutide in Persons with Obesity and Knee Osteoarthritis. N. Engl. J. Med..

[142] Weimann A., Braga M., Carli F., Higashiguchi T., Hübner M., Klek S., Laviano A., Ljungqvist O., Lobo D.N., Martindale R.G. (2025). ESPEN practical guideline: Clinical nutrition in surgery. Clin. Nutr..

[143] Santa Mina D., Clarke H., Ritvo P., Leung Y.W., Matthew A.G., Katz J., Trachtenberg J., Alibhai S.M. (2014). Effect of total-body prehabilitation on postoperative outcomes: A systematic review and meta-analysis. Physiotherapy.

[144] Carli F., Zavorsky G.S. (2005). Optimizing functional exercise capacity in the elderly surgical population. Curr. Opin. Clin. Nutr. Metab. Care.

[145] Ljungqvist O., Scott M., Fearon K.C. (2017). Enhanced Recovery After Surgery: A Review. JAMA Surg..

[146] Joint British Diabetes Societies (JBDS) for Inpatient Care (2016). Management of Adults with Diabetes Undergoing Surgery and Elective Procedures. https://abcd.care/joint-british-diabetes-societies-jbds-inpatient-care-group.

[147] (2022). NCT05546541. NUTRIRISK/REGAIN: Nutritional Status, Orthobiologic Quality, and Clinical Outcomes. NCT05546541.

[148] Meerwaldt R., Lutgers H.L., Links T.P., Graaff R., Baynes J.W., Gans R.O., Smit A.J. (2007). Skin autofluorescence is a strong predictor of cardiac mortality in diabetes. Diabetes Care.

[149] Yamagishi S., Fukami K., Matsui T. (2015). Evaluation of tissue accumulation levels of advanced glycation end products by skin autofluorescence. Int. J. Cardiol..

